# Enhanced North Pacific deep-ocean stratification by stronger intermediate water formation during Heinrich Stadial 1

**DOI:** 10.1038/s41467-019-08606-2

**Published:** 2019-02-08

**Authors:** X. Gong, L. Lembke-Jene, G. Lohmann, G. Knorr, R. Tiedemann, J. J. Zou, X. F. Shi

**Affiliations:** 1 Alfred-Wegener-Institut Helmholtz-Zentrum für Polar- und Meeresforschung, Bussestr. 24, 27570 Bremerhaven, Germany; 2 MARUM-Center for Marine Environmental Sciences, University Bremen, Leobener Strasse, 28359 Bremen, Germany; 3 Laboratory for Marine Geology, Qingdao National Laboratory for Marine Science and Technology, Qingdao, 266061 China; 4 Key Laboratory of Marine Sedimentology and Environmental Geology, First Institute of Oceanography, Ministry of Natural Resources, Qingdao, 266061 China

## Abstract

The deglacial history of CO_2_ release from the deep North Pacific remains unresolved. This is due to conflicting indications about subarctic Pacific ventilation changes based on various marine proxies, especially for Heinrich Stadial 1 (HS-1) when a rapid atmospheric CO_2_ rise occurs. Here, we use a complex Earth System Model to investigate the deglacial North Pacific overturning and its control on ocean stratification. Our results show an enhanced intermediate-to-deep ocean stratification coeval with intensified North Pacific Intermediate Water (NPIW) formation during HS-1, compared to the Last Glacial Maximum. The stronger NPIW formation causes lower salinities and higher temperatures at intermediate depths. By lowering NPIW densities, this enlarges vertical density gradient and thus enhances intermediate-to-deep ocean stratification during HS-1. Physically, this process prevents the North Pacific deep waters from a better communication with the upper oceans, thus prolongs the existing isolation of glacial Pacific abyssal carbons during HS-1.

## Abstract (web summary)

The role of the Pacific Ocean during the last deglacial is less well known. Here the authors used a complex Earth System Model and found that enhanced intermediate-to-deep ocean stratification, and an isolated carbon pool in the deep North Pacific during the last deglaciation when considered alongside proxy records.

## Introduction

The last deglacial atmospheric CO_2_ rise has been attributed to the release of deep, sequestered carbon out of the global ocean, likely linked to abrupt, millennial-scale changes of the Atlantic Meridional Overturning Circulation (AMOC)^1^. In particular, the rapid atmospheric CO_2_ rise during the AMOC off-mode in Heinrich Stadial 1 (HS-1, 17.5-14.9 ka) is thought to be mainly induced by the CO_2_ outgassing from the Southern Ocean^2–5^ and a lower efficiency of the biological carbon pump globally^6,7^. In parallel, studies have indicated a large storage of dissolved inorganic carbon (DIC) in the North Pacific deep waters (NPDW, the water mass below ~2500 m depth) during the Last Glacial Maximum (LGM, 23-18 ka)^8^. However, studies based on various marine proxies gave contradicting evidence that deep-ocean carbon was released from the North Pacific during HS-1 or, in contrast, remained isolated in a deep marine reservoir^8–13^. Specifically, such conflicting views revolve around whether or not the subarctic Pacific ventilation in HS-1 became deep and intense enough to connect to the deep ocean^9,14,15^. Once convection down to the deep ocean is established, the subarctic Pacific ventilation will be able to exchange properties, such as deep-ocean DIC, into intermediate depths, and then ventilate to the surface ocean for a potential CO_2_ release. Thus, the abrupt change of deglacial subarctic Pacific ventilation and the corresponding deep-ocean stratification are the two physical characteristics that determine the role of North Pacific Ocean in the global carbon cycle system during the last glacial termination.

**Fig. 1:**
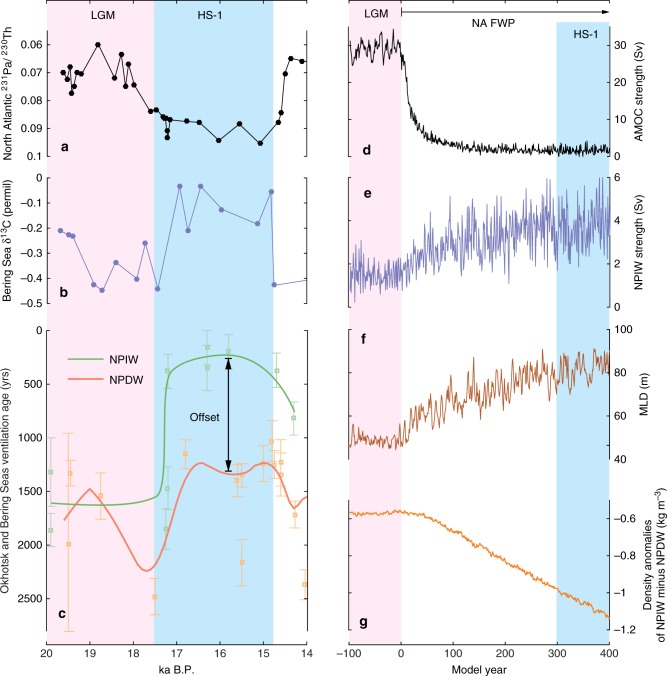
Collected proxy records and the modelling results in this study. **a** Reconstructed AMOC change on the basis of sedimentary ^231^Pa/^230^Th ratio;^3^ **b** Benthic foraminiferal δ^13^C records at the intermediate depths of the Bering Sea, with higher values for stronger NPIW formation;^15^ **c** NW Pacific intermediate and deep-ocean ventilation ages, with the error bars from radiocarbon measurements;^15^ **d** Modelled AMOC strengths; **e** Modelled NPIW strengths; **f** Modelled annual mean MLD in the middle of Okhotsk and western Bering seas; **g** Modelled subarctic Pacific NPIW (at 1000 m) and NPDW (at 3000 m) density anomalies

Under modern climate conditions, the North Pacific ventilation mainly occurs in the Okhotsk Sea and produces the North Pacific Intermediate Water (NPIW), dominantly controlled by brine rejection during winter sea ice growth^16,17^. Accordingly, paleoceanographic studies have hypothesized a role of potential sea ice expansion in causing a deeper ventilation (Fig. 1a–c) and probably stronger NPIW formation in HS-1 than during the LGM^15,18–20^, besides a direct control of a colder surface ocean^21^. In parallel, modelling studies have ascribed the stronger NPIW or NPDW formation of HS-1 to either intensified northward advection of warm, saline waters from the subtropical to the subarctic Pacific Ocean^14^, a removal of the subpolar North Pacific halocline^22^ or a colder and more saline surface subarctic Pacific Ocean during the North Atlantic cold events^23,24^. Nevertheless, a common characteristic of these otherwise partially exclusive explanations involves a breakdown of the surface-ocean density stratification in the subarctic Pacific Ocean. Regarding the maxima depths of deglacial North Pacific ventilation, there is hypothesized overturning throughout intermediate depths and down to the deep ocean of ~3000 m^11,14^. In contrast, some other studies have argued that the deep-ocean water is decoupled from the mid depths (~2000 m) and thus a continued isolation of the NPDW during HS-1^9,15,25,26^.

In this study, we use a complex Earth System Model, Max-Plank-Institute Earth System Model (MPI-ESM)^27^ (see Methods), to simulate deglacial North Pacific ventilation. Our work aims to complement the limitations of currently available proxy evidence in identifying the paleo-physical mechanisms of the HS-1 intensified North Pacific ventilation and development of North Pacific deep-ocean stratification. Our modelling results provide evidence for stronger NPIW formation during HS-1, triggered by increased surface salinity and strengthened by a positive temperature feedback. In conjunction with paleoceanographic evidence, we suggest an enhanced NPIW-to-NPDW stratification due to stronger NPIW formation during HS-1.

## Results

### Enhanced NPIW formation during HS-1

**Fig. 2:**
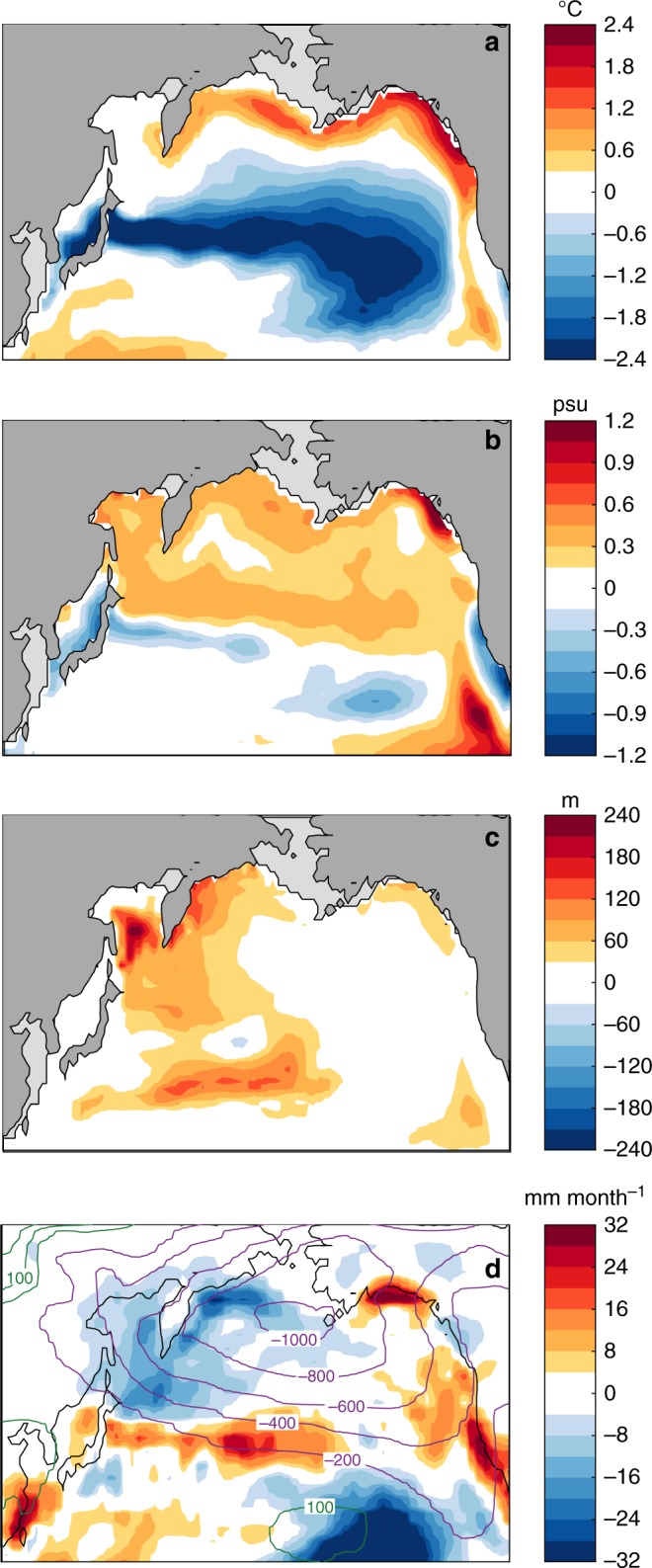
Modelled North Pacific change due to AMOC slow-down. **a** Winter SST anomalies of HS-1 to the LGM; **b** The coeval winter SSS anomalies; **c** The coeval wMLD anomalies; **d** The coeval change in winter atmospheric circulation. In **d**, the shading colours show net precipitation change, and the green and purple lines describing positive and negative change in sea level pressures, respectively. Here, the negative values of sea level pressure change over the subarctic Pacific Ocean imply an intensified Aleutian Low system in HS-1

In our HS-1 experiment (see Methods), when the North Atlantic freshwater perturbation (FWP) starts, the AMOC slows down rapidly, while the North Pacific ventilation gradually intensifies (Fig. 1d, e and Supplementary Fig. 1). In the following, we define the averaged state of 301-400 model years as the HS-1 state. It yields a quasi-equilibrium state of the substantially weakened AMOC with less than 5 Sverdrup (Sv, 1 Sv = 10^6^ m^3^ s^−1^), in line with the indication by marine proxies^28,29^, and a sustained maximum of NPIW formation with ~3.8 Sv (see Methods for the calculation of AMOC and NPIW strengths). In parallel, a N–S ocean transect along 180°E exhibits significantly lower salinities down to 2300 m depths, compared to the LGM state (Supplementary Fig. 2). This indicates a stronger NPIW rather than NPDW formation, in line with paleoceanographic evidence^9,15^. Moreover, our simulated LGM and HS-1 states reveal deepened winter (i.e. mean of January, February and March) mixed layer depths (wMLD) in the middle of the Okhotsk and the western Bering Seas, compared to the rest of the subarctic Pacific Ocean and its marginal seas (Figs. 1f, 2c, see Supplementary Fig. 3 for the MLD seasonality change). This marks these two areas as the predominant glacial NPIW source regions in our modelling simulations, corroborating paleoceanographic evidence^18,30,31^.

Our experiments characterize the middle of the Okhotsk and the western Bering Seas by higher sea surface salinities (SSS) and deepened wMLD in HS-1 compared to the LGM state, while the winter sea surface temperatures (SSTs) in the same area are not colder than during the LGM (Figs. 1f, 2a–c). Here, we attribute the stronger NPIW formation of HS-1 to the enhanced convection in the middle of Okhotsk and the western Bering Seas, controlled by a weakened surface halocline superimposed on the cold glacial surface ocean.

**Fig. 3:**
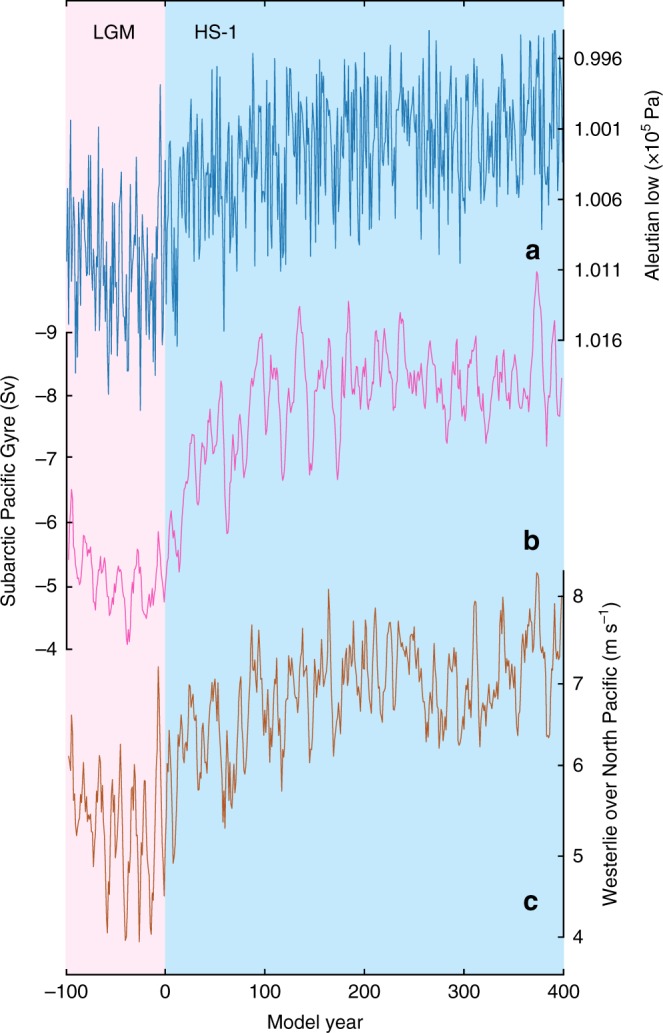
Modelled surface atmosphere and ocean change in the North Pacific climate system. **a** Strength of the Aleutian Low, indexed by the regional minima of sea level pressures; **b** Strength of the subarctic Pacific gyre, indexed by the regional minima in stream functions; **c**. Strength of the Westerlies averaged between 30 and 50°N over the North Pacific Ocean

In our results, the weakened surface halocline in the middle of the Okhotsk and western Bering Seas correlates with an intensified Aleutian Low-pressure system during HS-1. Once the AMOC weakens, the Aleutian Low intensifies simultaneously (Fig. 3a). This rapid atmospheric teleconnection from the North Atlantic to subarctic Pacific occurs via tropical latitudes with higher SSTs in the Eastern Equatorial Pacific acting as a pivot in the mechanism (Supplementary Fig. 4), as discussed in previous modelling studies^24,32^. Over the mid-latitude North Pacific, the stronger Aleutian Low causes stronger Westerlies and thus accelerates the North Pacific surface circulation (Fig. 3). As a consequence, larger amounts of subtropical-sourced, saline water are transported northwards, leading to the higher SSS in the subarctic Pacific Ocean and its marginal seas (Fig. 2b). Moreover, relative to the LGM, the strengthened Aleutian Low of HS-1 generates an anomalous cyclonic atmospheric circulation over the subarctic Pacific (Fig. 2d). This pattern transports drier air masses from the East Siberian continent to the Okhotsk and western Bering Seas. Such a process acts to additionally increase the SSS of the Okhotsk and western Bering Seas and thus weakens the surface halocline during HS-1 by reducing the regional net precipitation, in line with drier conditions in the Far East region during millennial-scale cold periods of the deglaciation as indicated by terrestrial proxies^33,34^. Furthermore, the anomalous cyclonic atmospheric circulation over the subarctic Pacific of HS-1 drives a stronger subarctic Pacific gyre at the AMOC off-mode (Fig. 3b). This results in a stronger upwelling of relatively warmer, more saline water from subsurface depths, by enhancing the Ekman pumping effect. In the surface ocean, the thermal buoyancy of these upwelled subsurface water is counteracted by a heat loss to the cold surface atmosphere; however, the water remains saline and thus further weakens the surface stratification. Overall, our results suggest that the intensified Aleutian Low of HS-1 weakens the surface halocline in the Okhotsk and Bering Seas via multiple processes in the atmosphere-ocean coupled system. Here, our results provide modelling support to previous hypotheses about the effective role of Aleutian Low in changing glacial NPIW formation, beyond earlier broad conceptual assumptions^20,35,36^.

In our results, the stronger convection in the Okhotsk and Bering Seas of HS-1 is initiated when the surface halocline becomes weakened while the SSTs in the same region are below 0 °C and similar to the LGM conditions. Therefore, our results suggest that the presence of a surface-ocean halocline prevents the NPIW from a higher formation during the LGM, although the cold winter SSTs should be conducive to a stronger NPIW production. This resembles the known low-salinity barrier that constrains the modern NPIW formation in the Okhotsk Sea^17^, and is also in line with previous modelling results about the dependence of glacial NPIW formation on the subpolar Pacific stratification^37^. Notably, our simulated stronger NPIW formation in HS-1 occurs on the basis of the continued existence of a halocline between the surface and intermediate ocean since the LGM, although such halocline becomes relatively weaker in HS-1 (Supplementary Fig. 2d–f). At the onset of the stronger NPIW formation during HS-1, the stronger convection transports more surface water with relatively lower salinities down to the intermediate-depth ocean, while it in turn ventilates the relatively more saline water from the intermediate to surface ocean. This process thus works to further weaken the halocline, acting as a positive feedback (salinity feedback, thereafter) for the NPIW intensification during HS-1. Here, our results indicate that a weakening, instead of a complete removal^22^, of the surface halocline triggers the stronger NPIW formation during HS-1 in conjunction with cold glacial surface ocean, and is also favoured by the salinity feedback.

Notably, our results show that the stronger northward transport of subtropical-sourced warmer and more saline surface water does not lead to a warmer surface ocean in the NW Pacific and its marginal seas during HS-1 (Fig. 2a), in line with previous paleoceanographic evidences^38,39^. In our results, the absence of a warmer NW Pacific in HS-1 is a result of the competition between atmospheric cooling impact and ocean warming potential. At the AMOC off-mode, the anomalous cyclonic atmospheric circulation of the stronger Aleutian Low transports a larger amount of cold air masses from the East Siberian continent to the Okhotsk and western Bering Seas. This process cools down the regional surface ocean, acting as the atmospheric cooling impact. In parallel, the oceanic warming potential correlates to the stronger northward advection of subtropical warmer water into the high latitudes North Pacific and the upwelling of relatively warmer water from the subsurface layer (due to the stronger ventilation and subarctic Pacific gyre). In the NW Pacific and its marginal seas, the atmospheric cooling impact and ocean warming potential work to change the SSTs in opposite ways, with their competition results determining the ultimate change in the SSTs from the LGM to HS-1. Here, these two competitive processes have important implication in understanding the distinct behaviours of the Atlantic-Pacific thermal seesaw in previous modelling studies: when the atmospheric cooling impact acts as the dominant control, the North Pacific shows lower SSTs in HS-1^31,40,41^; while when the ocean warming potential becomes in charge, the subarctic Pacific turns to warmer conditions against the cooling in the North Atlantic at the AMOC off-mode, thus constituting an Atlantic-Pacific thermal seesaw^37,42^. In this study, a case of close contest between the atmospheric cooling impact and oceanic warming potential results in the SSTs of HS-1 to be overall comparable with the LGM values in the Okhotsk Sea (Fig. 2a). This explains the same climatic signals in the paleo temperature reconstructions from this region^38,39^. Moreover, our modelling results characterize a non-uniform SST change in the vast area of the subarctic Pacific and its marginal seas from the LGM to HS-1. This is attributed to the spatial inconsistence of the competing amounts of the atmospheric cooling impact vs. oceanic warming potential. Here, our mechanism provides a unifying explanation for the regional heterogeneity among the previous paleoceanographic upper-ocean temperature reconstructions in the NW Pacific and its marginal seas^36,43^. In essence, the Atlantic-Pacific thermal seesaw is not a uniform feature of the high latitudes North Pacific in response the AMOC slow-down according to our modelling results, thus regionally decoupling it from the Atlantic-Pacific MOC seesaw^42^.

### Enhanced intermediate-to-deep ocean stratification during HS-1

**Fig. 4:**
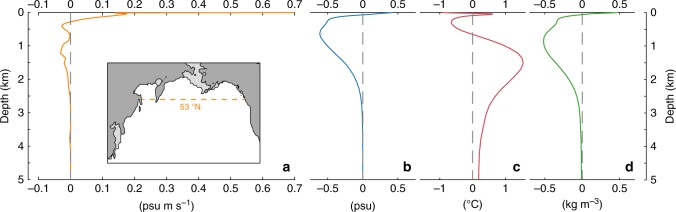
Modelled anomalies in the vertical profiles of the North Pacific Ocean and its marginal seas. **a** Northward salt flux averaged at 53°N across the entire North Pacific basin. The salt flux is calculated by multiplying salinity and meridional velocity, with the northward flux in positive values (see change in salinities and velocities at 53°N in Supplementary Fig. 5). **b**–**d** show the anomalies of the salinity, temperature and density averaged in the Okhotsk and west Bering Seas of the modelled HS-1 compared to LGM state, respectively

Our modelling results characterize the NPIW by lower densities in HS-1 compared to the LGM, meanwhile the NPDW shows only insignificant change (Fig. 4 and Supplementary Fig. 2). This leads to a larger contrast in the vertical density gradients and thus a strengthened NPIW-to-NPDW stratification during HS-1 (Fig. 1g). More specifically, our results reveal that lower densities of NPIW in HS-1 are attributed to its lower salinities and higher temperatures compared to the LGM (Fig. 4b–d). When the AMOC weakens in our experiment, a stronger halocline between the intermediate and deep ocean develops due to the salinity feedback. This constrains the impact of relatively colder deep ocean water on the intermediate depths by limiting the vertical water exchange, thus retaining the warmer characteristic within NPIW during HS-1 (Fig. 4 and Supplementary Fig. 2). In turn, this process also enhances the deep ocean stratification by enhancing the thermocline between the intermediate and deep ocean, thus creating a positive feedback (temperature feedback, thereafter) in developing an intensified intermediate-to-deep ocean stratification during HS-1.

In our results, the NPIW change from the LGM to HS-1 is characterized not only in the subarctic Pacific, but is also advected southwards via the mid-depth ocean circulation. This thus provides lower salinities and higher temperatures to the lower latitudes (Supplementary Fig. 5). In Fig. 4a, W-E transect at 53°N across the entire North Pacific Ocean shows a stronger-than-LGM southward transport of low-salinity water at intermediate depths during HS-1. This is attributed to the coeval stronger southward ocean flow mainly between 500 and 2000 depths (Supplementary Fig. 5b, f, j), thus it depicts a stronger advection of NPIW to lower-latitude Pacific during HS-1. Along the coast of the Gulf of Alaska, our results characterize a relatively warmer surface ocean, and a warmer and less saline intermediate ocean during HS-1 compared to the LGM (Supplementary Fig. 5d, f). These are in line with previously reported paleoceanographic evidence based on oxygen isotopes from foraminifera in this area^44,45^.

To assess the sensitivity of our results to the amount of North Atlantic FWP, we also performed additional experiments with a half (0.4 Sv) or twice (1.6 Sv) the strength of the freshwater fluxes. In these experiments, our results give the same qualitative results of the enhanced NPIW-to-NPDW stratification along with the stronger NPIW formation under the HS-1 climate conditions (Supplementary Fig. 6). In these two alternate experiments, a stronger North Atlantic FWP indeed leads to stronger NPIW formation and a further development of the NPIW-to-NPDW stratification, due to the correspondingly intensified Aleutian Low (Supplementary Fig. 7). Moreover, a reanalysis of previous modelling results^46^ using a different Earth System Model also suggested a similar connection between the NPIW intensification and NPIW-to-NPDW stratification development at AMOC off-mode, although the previous modelling results presented a higher sensitivity to the North Atlantic FWP (Supplementary Fig. 8). Therefore, we demonstrate that the development of North Pacific deep ocean stratification along with stronger NPIW formation is independent from the AMOC slow-down process, as long as the AMOC reaches an off-mode state. Furthermore, we have performed an additional experiment with a stepwise increase in FWP (Supplementary Fig. 9). This experiment shows that the North Pacific responses to the AMOC off-mode is robust for basic LGM AMOC background states that can be weaker or stronger than in our simulated pre-industrial (PI) state. These background strengths are comparable to other estimates for the LGM AMOC states^47,48^, although much weaker basic states^49^ cannot be excluded.

Our North Atlantic FWP experiments apply the LGM level of 193 ppm CO_2_, thus lower than the recorded HS-1 atmospheric CO_2_ increase in the ice cores^1^. To avoid an unintended impact of the higher CO_2_ during HS-1 to our proposed physics, we additionally conducted an experiment applying the 0.8 Sv FWP together with a 240-ppm atmospheric CO_2_ concentration. In such experiment, although the NPIW production rate becomes relatively lower than that under the 193 ppm CO_2_ conditions, it remains significantly stronger than the LGM conditions, while the AMOC also remains in an off-mode of less than 5 Sv (Supplementary Fig. 6). Thus, we argue that the AMOC slow-down exerts a decisive control on the physical response of the stronger NPIW formation and corresponding intermediate-to-deep stratification, aligning with the concept of Atlantic-Pacific MOC seesaw^9,15,42^.

**Fig. 5:**
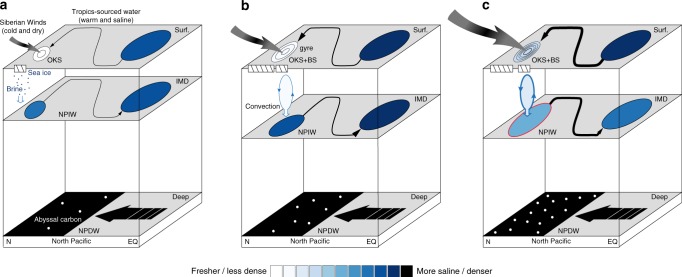
Schematic illustration of the mechanism proposed in this study and its implication for the reservation of North Pacific deep-ocean carbon. **a** The Modern; **b** LGM and **c** HS-1. OKS and BS mean the Sea of Okhotsk and Bering Sea, respectively. The white-bluish-black colours indicate an increase in salinities and also densities. In the subarctic Pacific region, denser circles indicate a stronger gyre circulation. In **c**, the red circle of the NPIW water indicates relatively warmer conditions during HS-1 compared to the LGM conditions

Our deglacial North Atlantic FWP experiments with a complex Earth System Model show a stronger NPIW formation and coeval enhancement of NPIW-to-NPDW stratification during HS-1. In our results, the NPIW formation maxima during HS-1 are attributed to the intensified North Pacific convection in the middle of Okhotsk and western Bering Seas, controlled by a significantly weakened surface halocline in conjunction with cold glacial SSTs and favoured by a salinity feedback. This stronger NPIW formation of HS-1 subducts a large amount of surface relatively less saline water down to the intermediate depths, as schematically illustrated in Fig. 5. This process determines the lower densities of the NPIW during HS-1. In parallel, our results yield no significant changes in NPDW densities from the LGM to HS-1. By enlarging the vertical density gradient, this results in an enhanced NPIW-to-NPDW stratification during HS-1. As a consequence, a deep-ocean thermocline develops due to constrained impact of the relatively colder deep ocean to the intermediate depths. In turn, such a deep-ocean thermocline also contributes to the development of the NPIW-to-NPDW density stratification during HS-1, acting as a positive feedback.

Our modelling results provide the physical paleoceanographic mechanisms to explain earlier hypotheses that postulates a stratification development between the NPIW and NPDW during HS-1, as shown in Fig. 1c. In these marine proxy studies, the NW Pacific ventilation age decreased at intermediate depths^15,50^, while it showed insignificant rejuvenation in the deep ocean during HS-1^15,25^. Here, our modelling results suggest that this is attributed to a physical control of the enhanced surface-to-intermediate ocean ventilation while the enhanced intermediate-to-deep ocean stratification during HS-1.

On a broader scale, our modelling findings have implications for scenarios of the North Pacific deep-ocean CO_2_ release during the last deglaciation, by showing constrained NPDW formation in response to the AMOC off-mode in HS-1. The inferred physical changes in the configuration of the North Pacific intermediate ocean overturning would significantly enhance and prolong any existing isolation of glacial Pacific deep waters and foster the persistence of a deep pool of sequestered carbon, which was ultimately released from the ocean to atmosphere in other locations^12,51^. As we hypothesize that NPIW and NPDW salinities are key factors in controlling the deglacial ocean ventilation, paleo-salinity reconstructions in future proxy studies would be desirable to further develop the understanding of the physical determinants of the Pacific deep-ocean stratification and carbon pool evolution, in particular during past deglacial warming episodes.

## Methods

### Earth System Model MPI-ESM

In this study, we use the fully coupled Max-Plank-Institute Earth System Model (MPI-ESM, version 1.2.00p4)^27^. It employs the atmospheric model ECHAM6^52^ with the land surface component JSBACH^53^ at T63 resolution (1.875° × 1.875°) and 47 vertical layers. In addition, the MPI-ESM uses Max-Plank-Institute Ocean Model (MPIOM)^54^ as the ocean component. It has the GR15 (~1.4° × 0.8°) horizontal resolution and 40 uneven vertical layers, and applies the sea ice dynamics of viscous-plastic rheology^55^. The model has been previously used for both glacial and interglacial paleoclimate studies^27,56^ and a comparable model version has been employed for the Coupled Model Intercomparison Project Phase 5 (CMIP5)^57^.

### Experimental design

Before simulating the HS-1 climate, we firstly conducted an experiment for the LGM conditions to a quasi-equilibrium state. Our LGM experiment simulates for 4000 model years until a quasi-equilibrium state with SST change less than 0.05 °C per 100 years and no trend in the variability of the AMOC strength. Here, our LGM experiment applies the full set of the Paleoclimate Modelling Intercomparison Project Phase III (PMIP3) experimental setup for the LGM, including the 21 ka astronomical parameters, greenhouse gases, ice sheets and the corresponding lowered sea level (see http://pmip3.lsce.ipsl.fr/ for the details). Our LGM experiment results show a shallower, but relatively stronger AMOC of ~28 Sv, compared to the ~ 24.5 Sv AMOC in the PI, control state. On the basis of the LGM ocean, we mimic the HS-1 climate conditions by applying an 0.8 Sv FWP^58^ to the North Atlantic Ice-Rafted Debris belt region (40°N–55°N, 45°W–20°W)^59,60^. We conduct the FWP experiment for 400 years until a quasi-equilibrium states, when both the AMOC and NPIW strengths show insignificant trends, respectively (Fig. 1d, e).

### Calculation of AMOC and NPIW strengths

In our experiments, the calculation of AMOC strength refers to the maximum value of the Atlantic Ocean stream function in the upper 200–3000 m depths and 30°N northward^46,61^. In addition, the NPIW (or NPDW) strength is calculated by the maximum value of meridional overturning stream function in the Pacific of 40–70°N, 0–3500 m depth (Supplementary Fig. 1).

### Response of the NW Pacific sea ice to off-mode AMOC

Studies have shown that the response of stronger NPIW formation to a off-mode AMOC is attributed to various mechanisms on the basis of the LGM state^15,18–24^. In parallel, along with the stronger NPIW formation of HS-1, the NW Pacific are characterized by comparable-to-LGM SSTs in the Okhotsk Sea during HS-1^38,39^, suggesting uncertainties in sea ice response to the stronger NPIW formation along with the large sea ice coverage under maxima glacial conditions (Supplementary Fig. 10), thus distinct from the modern climate^16,17^. In our modelling results, the sea ice concentration is characterized by relatively lower values in the southern part of the Sea of Okhotsk and the Bering Sea. On the other hand, the sea ice edge in our FWP experiment with 0.8 Sv is in line with the HS-1 sea ice area indicated by palaeoceanographic evidences^62,63^, as shown in Supplementary Fig. 11.

### Independence of NPIW intensification of HS-1 from LGM AMOC

Paleoclimate modelling studies have presented diverse results in term of the AMOC strength of the LGM compared to the PI conditions^64,65^. In this study, our simulation of the LGM climate has the AMOC of ~28 Sv, stronger than the ~24.5 Sv in the PI control experiment (Supplementary Fig. 1). Here, we also included an experiment with a 0.2 Sv FWP firstly and then a stronger hosing of 0.8 Sv. As shown in the Supplementary Fig. 1e and Supplementary Fig. 9, the 0.2 Sv FWP weakened the AMOC from the LGM ~28 Sv to a quasi-equilibrium state of ~20 Sv. Here, we use the LGM state (28 Sv AMOC) and LGM with 0.2 Sv FWP (20 Sv AMOC) as the representatives for the LGM with stronger- and weaker-than-PI AMOC background, respectively. In this new experiment, the NPIW strength showed insignificant change when the AMOC slowed down to the 20 Sv due to the 0.2 Sv FWP, compared to the LGM state of ~28 Sv (Supplementary Fig. 1f and Supplementary Fig. 9b). On the other hand, when the following 0.8 Sv FWP collapsed AMOC to an off mode (less than 5 Sv), the NPIW rapidly increased to ~2.8 Sv. Therefore, the NPIW response to the off-mode AMOC based on the LGM state with 0.2 Sv FWP (i.e. the case for the LGM with weaker-than-PI AMOC background) is in line with the NPIW intensification due to the AMOC collapse based on the LGM state of a stronger-than-PI AMOC (Fig. 1d, e and Supplementary Fig. 9b). Accordingly, although based on a single model, our results indicated that the response of the NPIW intensification to the collapsed AMOC during HS-1 is independent from the LGM AMOC background.

### Code availability

The MPI-ESM climate model codes are available by a registration at http://www.mpimet.mpg.de/en/science/models/license/, and the scripts used to generate the plots in this paper are available from the corresponding author on request.

## Supplementary Information

Supplementary Information

Peer Review File

## Data Availability

All relevant data in this paper have been uploaded to PANGAEA Data Publisher https://issues.pangaea.de/browse/PDI-19613.
