# Supplementary material for: Enhanced North Pacific deep-ocean stratification by stronger intermediate water formation during Heinrich Stadial 1
Source: Nat Commun. 2019 Feb 8;10:656. doi: 10.1038/s41467-019-08606-2 (PMC6368553; doi:10.1038/s41467-019-08606-2)
Supplement: Supplementary file 1 — Supplementary Information [file 41467_2019_8606_MOESM1_ESM.pdf]

-- Supplementary Information --

Enhanced North Pacific deep-ocean stratification by stronger intermediate water formation during Heinrich Stadial 1

Gong et al.

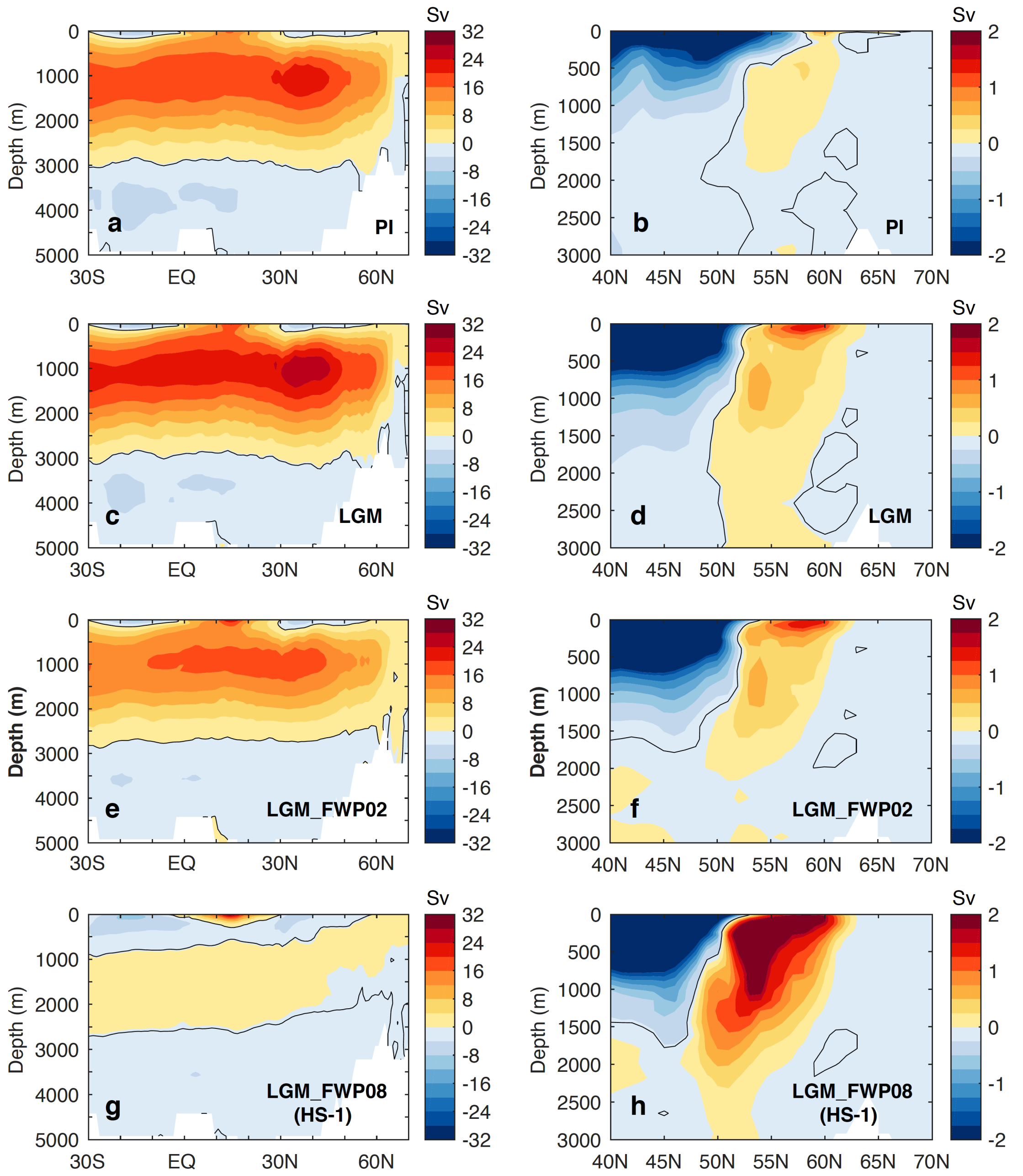

Supplementary Fig. 1 Modelled AMOC and NPIW sections. a. AMOC of the PI; b. NPIW of the PI; c. AMOC of the LGM; b. NPIW of the LGM; e. AMOC of the LGM 0.2Sv-FWP experiment, as a representative of an LGM state with weaker-than-PI AMOC; f. NPIW in the LGM 0.2Sv-FWP experiment; g. AMOC of the modelled HS-1 conditions; h. NPIW of the modelled HS-1 conditions.

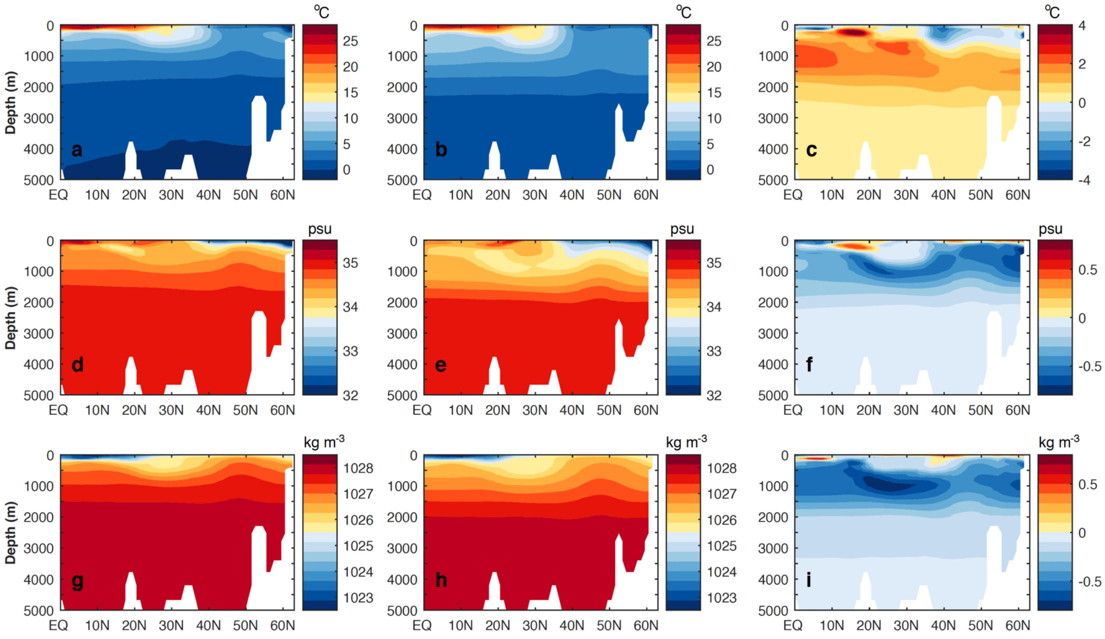

Supplementary Fig. 2 Modelled temperature (T), salinity (S) and density (D) change at 180°E. a, d and g show the T, S, D under the LGM conditions, respectively. In comparison, b, e and h present the T, S, D in the HS-1 experiment, respectively. Additionally, c, f and i reveal the T, S and D anomalies in HS-1 compared to the LGM conditions. As shown, the low SSS is critical in maintaining the vertical density stratification during the LGM, as it is observed in modern physical oceanographic observations[64, 65].

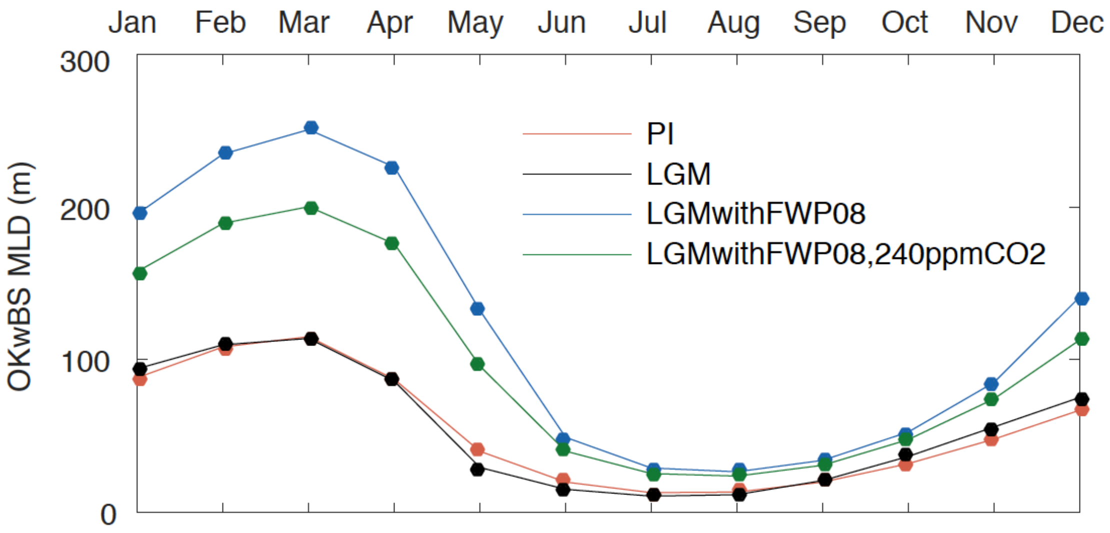

Supplementary Fig. 3 Modelled seasonality of the MLD averaged over the Okhotsk and western Bering Seas, where the glacial NPIW forms. In our FWP experiments, once an application of atmospheric CO_2 of 240 instead of 190 ppm, although the winter MLD in the Okhotsk and Bering Seas become relatively shallower, they remain significantly stronger than the LGM conditions.

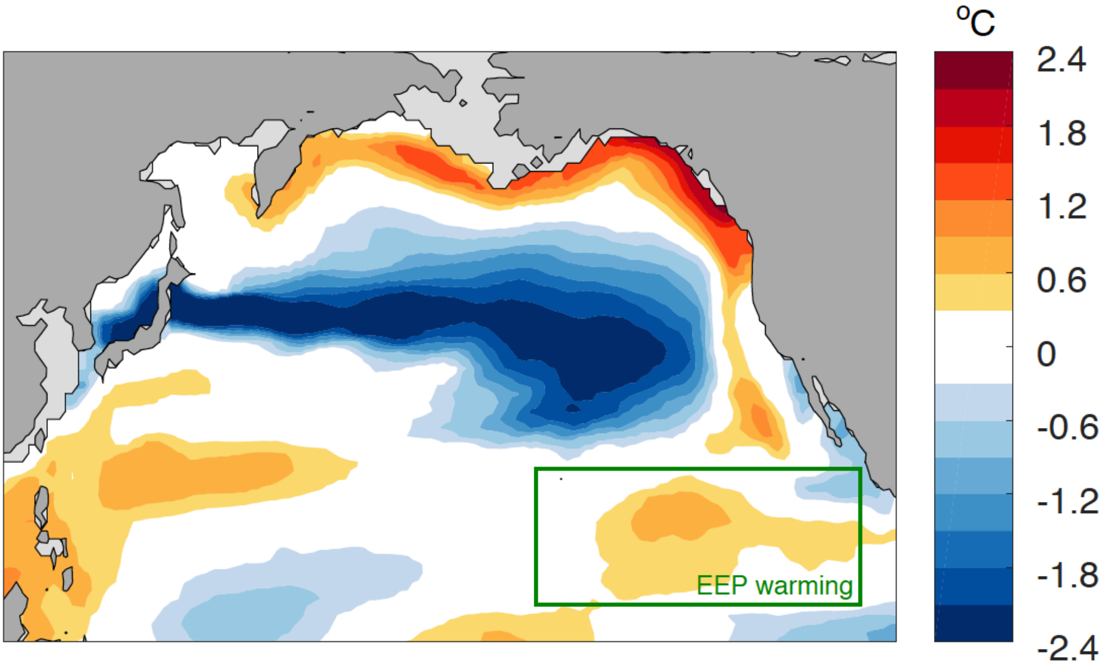

Supplementary Fig. 4 Modelled North Pacific winter SST anomalies due to the AMOC slow-down. The green rectangular box highlights the higher SST in the East Equatorial Pacific (EEP), as the key pivot that resulted the stronger Aleutian Low once the AMOC weakened, thus in line with the mechanism in previous modelling studies[24, 32].

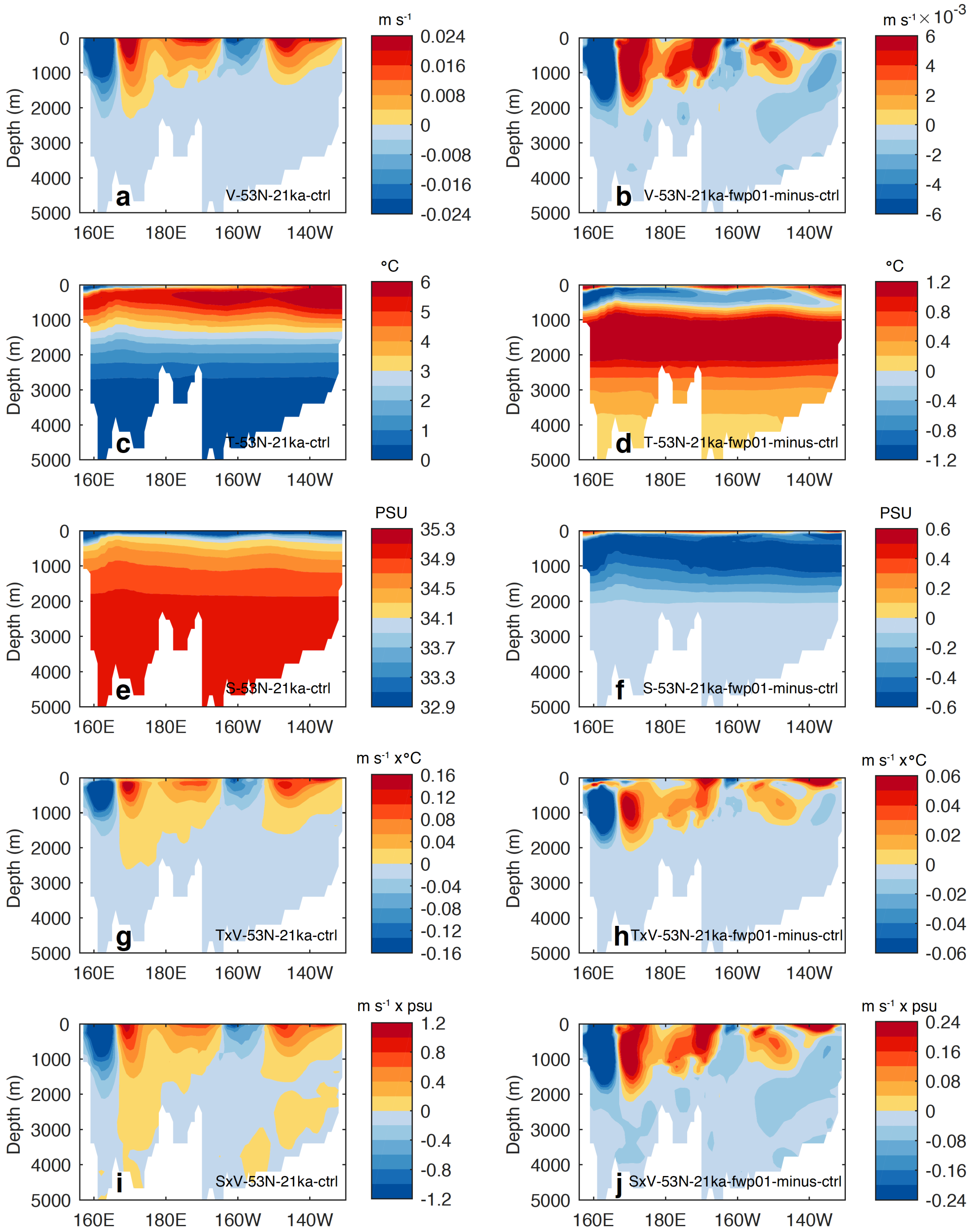

Supplementary Fig. 5 Modelled ocean state and its anomalies at 53°N across the entire North Pacific Ocean during HS-1 compared to the LGM conditions. a, c, e, g and i show the LGM-state Northward velocity, temperature, salinity, northward heat flux (a multiply of temperature and velocity) and salt flux (a multiply of salinity and velocity), respectively. The right column shows the HS-1-to-LGM anomalies at 53°N in the Gulf of Alaska of b. Northward velocity; d. Temperature; f. Salinity; h. Northward heat flux and g. Salt flux, respectively.

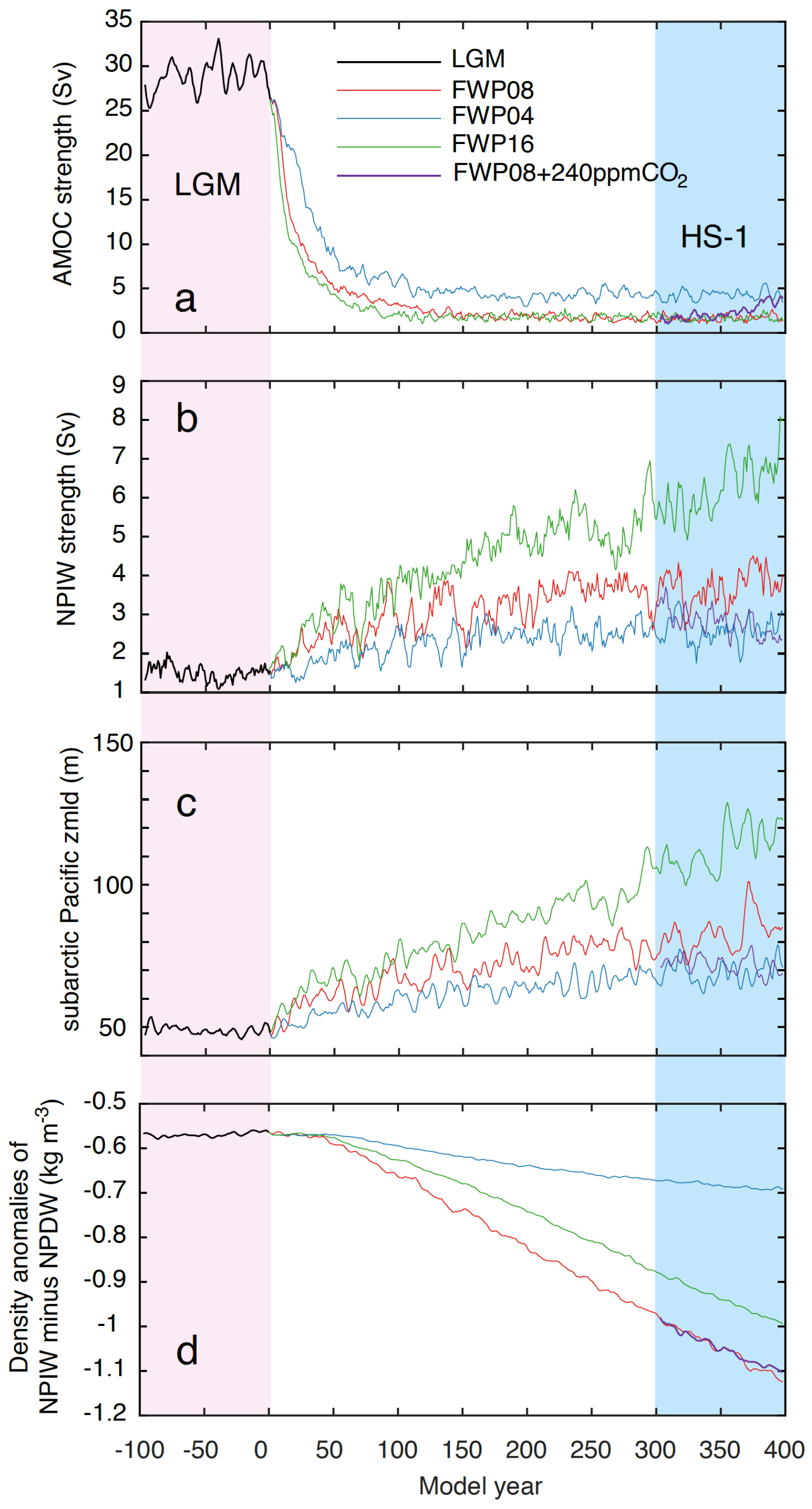

Supplementary Fig. 6 Common features in the FWP experiments of different strengths and one experiment also with the atmospheric CO_2 of 240 ppm. a. AMOC; b. NPIW; c. Annual mean MLD averaged over the subarctic Pacific Ocean; d. Subarctic Pacific NPIW (at 1000 m) and NPDW (at 3000 m) density anomalies.

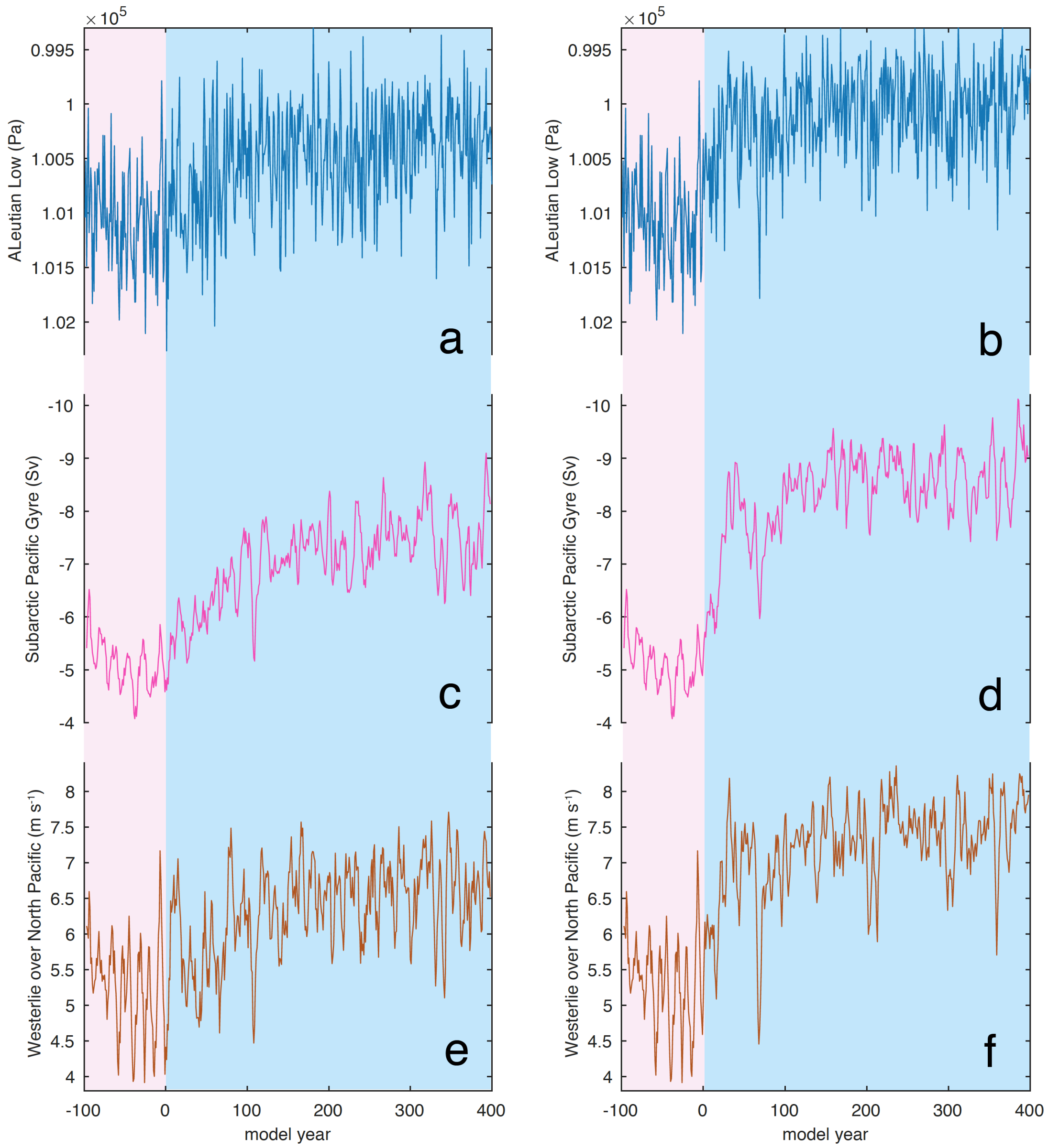

Supplementary Fig. 7 Modelled surface atmosphere and ocean change in the North Pacific climate system in the experiments with 0.4 and 1.6 Sv FWP. a, b. Strength of the Aleutian Low; c, d. Strength of the subarctic Pacific gyre; e, f. Strength of the Westerlies above the Pacific Ocean. The left and right columns show the results in the experiments with 0.4 and 1.6 Sv FWP, respectively.

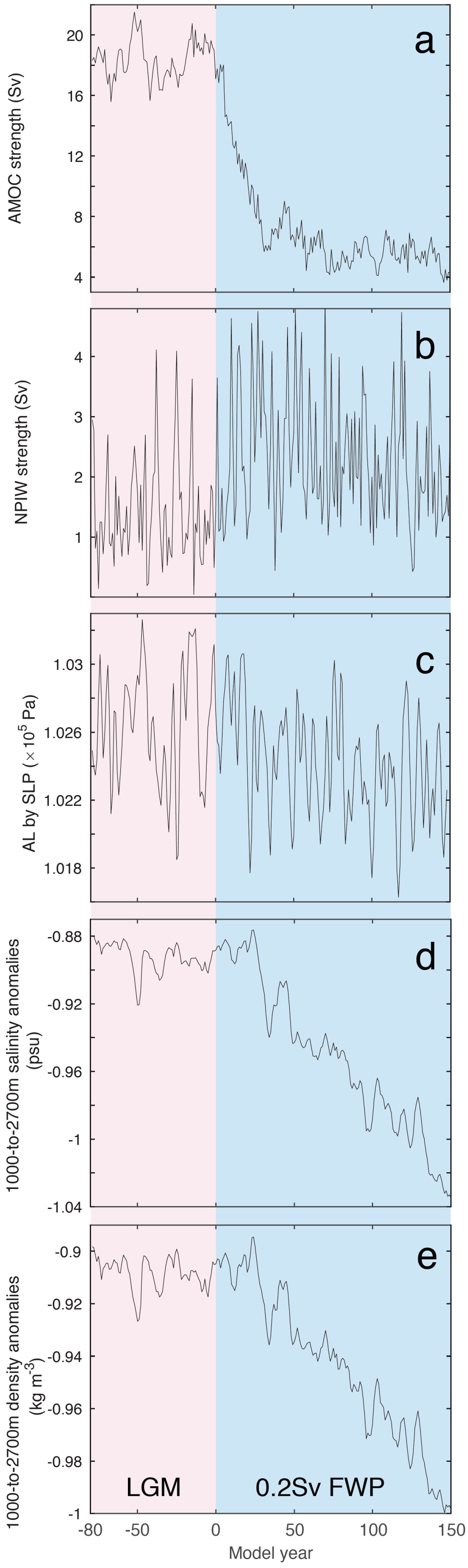

a
b
c
d
e
AMOC strength (Sv)
NPIW strength (Sv)
AL by SLP ($\times 10^5$ Pa)
1000-to-2700m salinity anomalies (psu)
1000-to-2700m density anomalies (kg m^{-3})
LGM
0.2Sv FWP
Model year

Supplementary Fig. 8 Reanalysis of the COSMOS-modelled North Pacific change of HS-1 compared to the LGM state. This LGM-based, 0.2Sv FWP experiment in the northern North Atlantic Ocean has been documented and analysed for the change in the North Atlantic Ocean in our previous work Gong et al. [2013][46]. In this figure, a. AMOC strength; b. NPIW strength; c. Aleutian Low index; d. the North Pacific intermediate-to-deep ocean halocline due to AMOC slow-down; e. The coeval North Pacific intermediate-to-deep ocean density stratification along with the AMOC slow-down. As shown, AMOC collapsed due to 0.2 Sv FWP (less than 5 Sv), suggesting different sensitivity to the North Atlantic FWP from the MPI-ESM modelling results in this study. On the other hand, despite the distinct sensitivity to the FWP compared to the MPI-ESM results, the COSMOS in a similar way revealed enhanced NPIW formation and the coeval development in the North Pacific intermediate-to-deep salinity and density stratifications in response to the collapse in AMOC. Together with our results using MPI-ESM, the reanalysis of the COSMOS-Modelling results suggests the dominant role of an AMOC 'off-mode' in determining the change in North Pacific Ocean during HS-1.

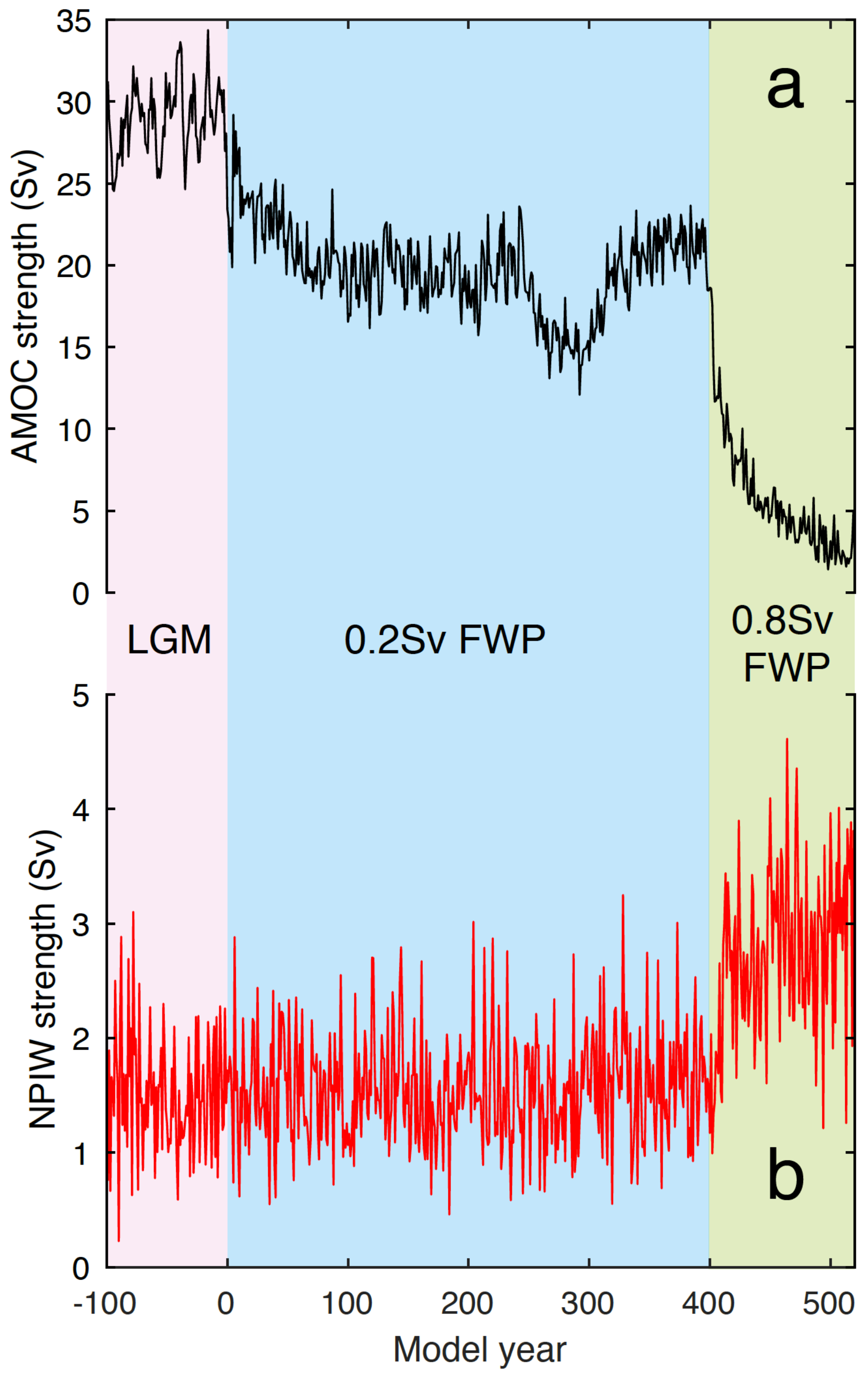

Supplementary Fig. 9 Modelled AMOC and NPIW strengths in the experiment with a 0.2Sv FWP for 400yrs firstly and then a stronger one of 0.8 Sv for another 120yrs. Here, we use the 0.2 Sv-hosed state of ~20 Sv AMOC as a representative for a LGM state with the AMOC weaker than PI conditions (~24.5 Sv for PI, see Supplementary Fig. 1). a. AMOC strengths; b. NPIW strengths.

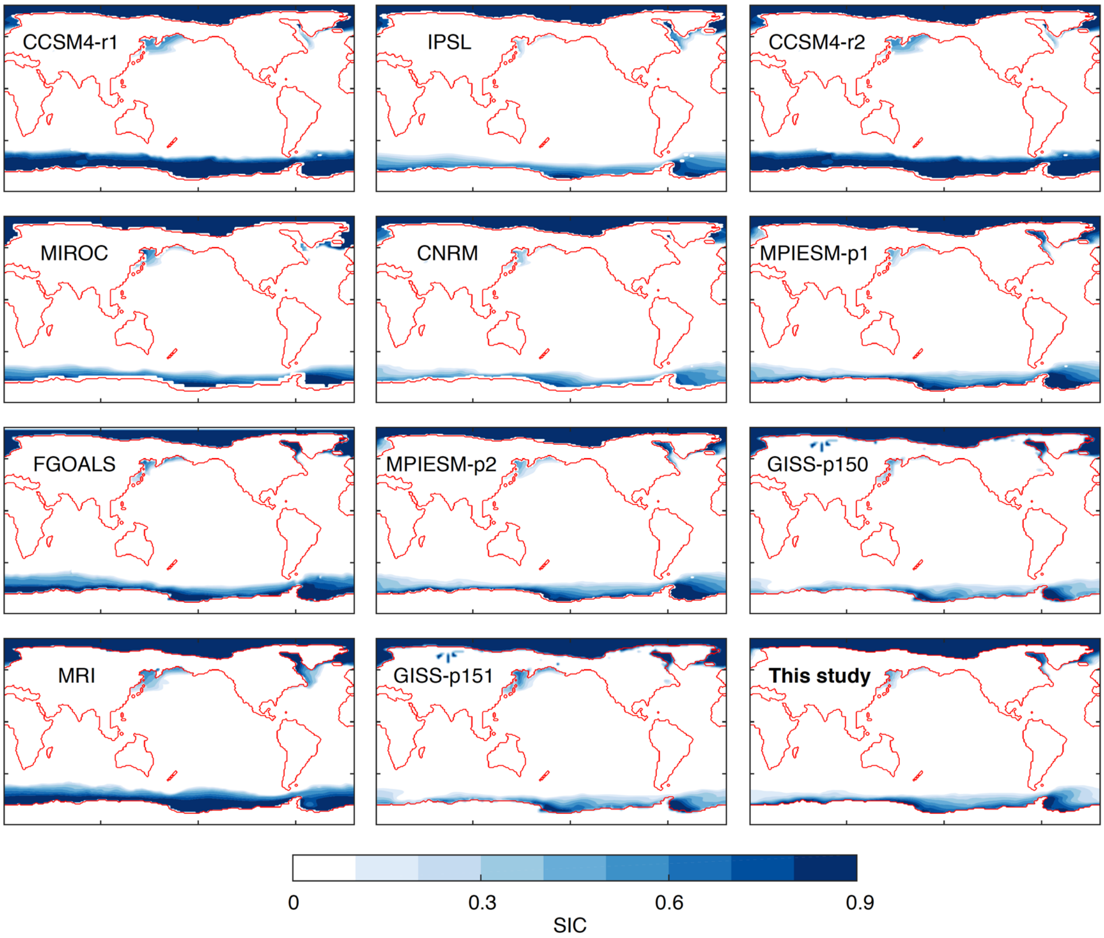

Supplementary Fig. 10 Modelled LGM sea ice fractions by different Earth System Models (*https://esgf-node.llnl.gov/search/esgf-llnl/*).

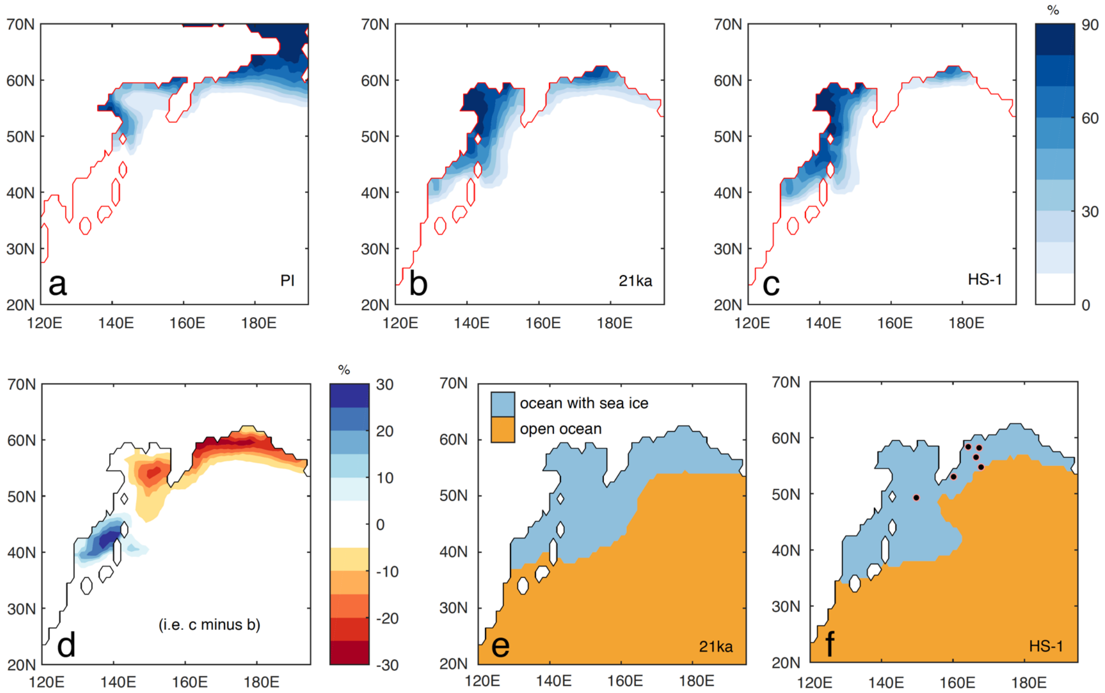

Supplementary Fig. 11 Modelled Sea ice and paleo sea ice reconstructions. a, b and c showed the modelled winter sea ice concentrations in the PI, LGM and HS-1 experiments, respectively. d. Modelled winter sea ice concentration anomalies of HS-1 to LGM conditions, i.e. c minus b. e and f show the sea ice expansion in the LGM and HS-1 experiments, respectively. In f, the circled black dots are PIP25-indated sea ice region during HS-1[62].
