# Supplementary material for: Enhanced North Pacific deep-ocean stratification by stronger intermediate water formation during Heinrich Stadial 1
Source: Nat Commun. 2019 Feb 8;10:656. doi: 10.1038/s41467-019-08606-2 (PMC6368553; doi:10.1038/s41467-019-08606-2)
Supplement: Supplementary file 2 — Peer Review File [file 41467_2019_8606_MOESM2_ESM.pdf]

Reviewer #1 (Remarks to the Author):

Gong et al. present an intriguing model study documenting changes in North Pacific Intermediate Water (NPIW) formation across HS1. This study addresses an important aspect in paleoceanography, which has been a matter of intense debate during the last decade or so.

The model outputs show enhanced NPIW formation during HS1 concomitant with a substantial reduction in North Atlantic Deep Water (NADW) formation associated with meltwater discharge, consistent with proxy data. Convection in the Sea of Okhotsk and the Bering Sea is enhanced as a result of increasing SSS associated with increased northward transport of saline subtropical waters. Enhanced NPIW formation leads to a strengthening of the intermediate-to-deep stratification, preventing CO2-rich abyssal waters to communicate with the atmosphere. These observations contradict previous proxy evidence calling for enhanced deep water formation during HS1. The authors further convincingly show that these observations are robust and are not dependent on the hosing intensity applied to slow down NADW.

I really enjoyed reading the manuscript, although the argumentation can be quite cumbersome in places. It advances our understanding of North Pacific paleoceanography, in particular related to its role in contributing to the deglacial increase in atmospheric CO2. The study has the sort of attributes to warrant publication in a broad audience journal such at Nature Communications. I have listed a number of comments below, which I would like to see addressed before I can recommend this manuscript to be accepted for publication. I hope the authors will find my comments helpful and constructive as they aimed to be.

* Please clearly define/quantify what is meant by “abyssal” (l. 14 and throughout), “deep” (l. 19 and throughout) and “intermediate” depths (l. 20 and throughout) as the loose/incoherent definition of these concepts has spurred unnecessary confusion within the community.

l. 36. This is certainly correct. Please also note that both model (e.g. Schmittner & Galbraith, 2008) and data compilation (Galbraith and Jaccard, 2015) indicate a general weakening of the global efficiency of the biological pump early in the deglaciation, which would have contributed to partition CO2 out of the ocean interior.

l.41-43. Unclear - please rephrase

l. 63-67. Unclear - please rephrase

l. 78. The term “suppressed” is confusing here. I would rather say that NADW was “substantially weaker” (consistent with paleoceanographic proxies (e.g. Bradtmiller et al., 2014 NComms; Lippold et al., 2016, EPSL)).

l. 84-89. Great! Strong argument in favor of the robustness of the model, highlighting its suitability to address the hypothesis outlined in the introduction.

l. 84-87. What are the implications of deeper mixed layer depths for vertical nutrient supply and export production? Proxy evidence in both the Sea of Okhotsk and the Bering Sea indicate lower export production during HS1 (e.g. Brunelle et al., 2010). Is this further evidence for a stronger decoupling between surface and nutrient-rich deep waters?

l. 121. This argument is consistent with proxy data from covering the entire subarctic Pacific gyre (Kiefer and Kienast, 2005)

l. 125 – would a stronger Aleutian Low (possibly related to larger North American ice sheets during HS1) increase Ekman pumping in the subarctic Pacific and its marginal seas? If true, this argument would again argue for a stronger decoupling between surface and nutrient-rich deep waters, as export production was consistently lower before the B/A throughout the subarctic Pacific (e.g. Galbraith et al., 2007; Kohfeld & Chase, 2011).

l. 130-131. Does this argument also take into account the upwelling of relatively warmer subsurface waters as a consequence of increased convection during HS1?

l. 132. Please also cite Kiefer and Kienast, 2005.

l. 136. Is there evidence for reduced river discharge during this time interval that would corroborate this argument?

l. 147-163. This argument is confusing and doesn’t make sense to me. You mention earlier that NPIW formation increases during HS1 as a result of increasing SSS (while maintaining approximately constant SSTs). As such, I don’t understand why NPIW densities decrease compared to the LGM, which is characterized by lower SSS once convection is initiated.

I understand that the model requires higher SSS in the Sea of Okhotsk and the Bering Sea to kick-start convection. Does this imply that saline water transported from the subtropical Pacific accumulates in both regions, before initiating convection? Why then SSS decrease upon initiation of enhanced NPIW formation? Is the northward transport of subtropical waters not efficient enough to

replace the volume of water, which has been subducted? In any case this paragraph needs to be clarified.

l. 168 and throughout. Use “Gulf of Alaska” instead of “Alaska Bay”.

Reviewer #2 (Remarks to the Author):

The authors present a modeling study of the early parts of the last deglaciation with a focus on North Pacific Ocean circulation and ventilation changes. The new idea is that enhanced intermediate water formation during HS-1 would advect relatively low salinity waters downward and thus decrease densities of intermediate waters, which would increase the density difference between intermediate and deep waters. I think the authors make a good point that this is a plausible scenario, but I also think that their conclusions and inferences are overconfident and therefore the paper needs major revisions.

I appreciate that the authors present sensitivity tests with respect to the amount of freshwater forcing applied to the North Atlantic and atmospheric CO2 concentrations. However, I think the results may also be critically dependent on uncertain initial conditions, which were not considered. E.g. Schmittner et al. (2007, VOL. 22, PA3207, doi:10.1029/2006PA001384) have shown that the response in the North Pacific to North Atlantic hosing depends critically on the background stratification. If the stratification is strong the response is much reduced compared to a weakly stratified initial stratification. Another sensitivity not considered is the initial (LGM) AMOC strength. Currently the LGM AMOC is unknown. Whereas some papers suggest a strong AMOC (Kurahashi-Nakamura et al., 2017, doi:10.1002/2016PA003001) others suggest a weak AMOC (e.g. Schmittner and Somes, 2016, doi:10.1002/2015PA002905). Thus, I think it is fair to conclude that there is no consensus and the LGM AMOC strength remains uncertain. In this context it would be appropriate to discuss the sensitivity of the results to the unknown LGM AMOC strength. E.g. if the LGM AMOC was weak, in contrast to the author’s model, the North Pacific response may also be different. I think a discussion of these uncertainties would be appropriate.

Previous model results also did not agree on the temperature response in the North Pacific. E.g. Mikolajewicz (1997) and Zhang and Delworth (2005) suggest cooling, whereas others (e.g. Schmittner et al. 2007) suggest warming. I think this also warrants some discussion. If I remember correctly I think Mikolajewicz (1997) suggest an atmospheric mechanism for the cooling, whereas Schmittner et al. (2007) suggest an ocean mechanism (the Atlantic-Pacific seesaw of Saenko et al.). I think the author’s experiments could add something to this issue.

98-99 why do NH westerlies intensify? Fig. 3a seem to also show a southward shift. Please comment in text. A map of surface wind or windstress vector anomalies may be useful to see where this intensification happens. I assume much will be over the North Atlantic. Results from Schmittner et al. (2007) indicate that the response in the North Pacific is highly model dependent.

100-101: “As a consequence a larger amount of tropically-sourced saline water is transported northward” This statement is not supported with evidence. How do you know that wind changes are the cause? In some of Schmittner et al.’s (2007) experiments and in Saenko et al. (2004) the North Pacific salinifies as a response to ocean buoyancy forcing only without any wind changes. So, in order to attribute northward salt transport changes to wind a decomposition into overturning and wind-driven gyre transport is needed. Or the statement needs to be changed. Also, how do you know it is “tropically-sourced”. I don’t see evidence provided for this either. Could it be subtropically-sourced? I’d think that would be more likely.

109: “)” corresponding opening parenthesis is missing

113-114 “wintertime” show seasonal SSS evolution somewhere to back up this statement with evidence

“extremely cold” SSTs are mentioned e.g. in lines 105, 108 and 205. What does that mean? SSTs can only be at most -2degC cold.

109-110 “that a presence of relatively stronger surface halocline in LGM (Supplementary Fig. 2)” stronger compared to what? The pre-industrial control? But this is not shown in Fig. S2. Again, evidence seems to be missing to support this statement. Also, an explanation as to why the halocline was stronger would be useful. Is it because of the closure of Bering Strait?

125-127 In Mikolajewicz (1997) and Zhang and Delworth (2005) atmospheric circulation changes lead to cooling. See also discussion in paragraph 18 of Schmittner et al. (2007). So, models don’t seem to show consistent responses in the North Pacific due to an AMOC shutdown.

129: “tropical” see above

144: “act” syntax; I suggest “act as”

169: I’m not sure I see a “stronger southward flux of lateral salt transport” in the figure. The figure shows positive and negative patterns. Please refer to specific features in the figure. Also, why only

show the eastern portion of the basin? Why not show the whole zonal section? Surely the western part also transports salt.

171: “stronger southward ocean flow” do you mean the in Fig. 5b between 130W and 140W? It would be helpful for the reader to point out specific features as precisely as possible, otherwise the reader is forced to guess what is meant.

172: “thus a depict” syntax

191 “HS-1, higher CO2” syntax; I suggest “higher CO2 during HS-1”

206 “and are” syntax

220 Since ventilation ages were not simulated attribution of their changes is not possible. This is speculation.

225-226 “rule out” I don’t think this can be ruled out. I think the authors present a physically consistent viable mechanism but because no real apples-to-apples data model comparison has been performed ruling out other scenarios is not possible.

Reviewer #3 (Remarks to the Author):

This manuscript presents interesting model results about how North Pacific stratification responded to circulation changes during Heinrich 1 with implications for better understanding the source and timing of carbon release from the deep ocean to the atmosphere during the deglaciation. This topic is actively debated among researchers and is likely to be of interest to a relatively broad audience. Hence, it may be suitable for publication in Nature Communications after revision.

I have several concerns about the manuscript’s current form that could be addressed with major revisions. Generally, the manuscript needs to provide more background about previous studies and more detail about the model results and uncertainties. Also, the manuscript needs revision to improve the writing and figure presentation.

Placing the new results in the context of previous research:

1. This manuscript should compare the new results with previous modeling efforts about this question (e.g., Chikamoto et al., 2012). How do the current modeling results compare with previous ones? How do differences between models potentially contribute to differences in the results?

2. Lines 132 and 145 claim that the new model results agree well with paleo-temperature reconstructions from proxies. The manuscript needs a figure (or supplemental figure) that presents a comparison of the proxy and model results.

Model details:

3. Line 54 lists sea ice expansion as a potentially important mechanism for affecting NPIW formation during HS-1. However, this appears to be the only mention of sea ice in the entire manuscript. The authors need to provide information about any sea ice changes that occurred in their model and how these did or did not contribute to NPIW formation change.

4. Additionally, the authors should discuss how their model compares with other Earth System model results for the pre-industrial and LGM (particularly with respect to overturning strength/depth and sea ice extent). Could model differences contribute to any bias in the results?

5. How long was the spin-up for the LGM run? What condition was used to determine when the LGM had reached a "quasi-equilibrium state"?

Writing and figure presentation:

6. There are quite a few sentences in this manuscript that are awkwardly or incorrectly phrased. The manuscript needs extensive revision with this in mind. For example, lines 41-45. A few other examples of word choice problems:

- Line 46-48: I wouldn't describe a change in ventilation as a "fundamental physical characteristic."
- Lines 64-66: "a hypothesized overturning ... CONTRASTS (instead of contrasted) with a decoupling …and CONTINUED (instead of remained) isolation …"
- Line 172: "thus a depict of stronger influence" ???
- Line 378: "radish colours present a stronger northward transport" should be "red colours represent …"

7. Because the abstract begins with a question about CO2 release from the North Pacific, it would be nice if it ended with a more definitive statement about when/how this CO2 release occurred (instead of only a statement about prolonged isolation of the deep Pacific). The manuscript addresses this in more detail but the abstract feels incomplete.

8. Figure 1f: I recommend flipping the y-axis to match the sense of depth increasing downward.

9. Figure 3: These are three unrelated subplots that shouldn't be grouped together. I recommend keeping only one of these subplots in the manuscript and changing the rest to supplemental figures.

10. Figure 4: I think the colors here illustrate an absolute scale for salinity/density, but the caption makes it sound like the colors indicate change relative to some reference: "… colors indicate an increase in salinities …"

11. It's confusing that Figures S1 (b, d and f) have a different y-axis range than S1 panels a, c, and e. It would also be nice if S1 (b, d and f) matched the same x and y axis ranges as Figure S2. Figures 3b and S5 could also match the vertical scale of these other plots.

A correction about the implementation of the North Atlantic freshwater perturbations that is stronger than the stated in the previous manuscript.

In this revision work, we found a calculation error in converting freshwater perturbation (FWP) amount to the freshwater flux into each model grid cell, resulting in eight-times stronger FWP than the stated in the previous manuscript. In the revised manuscript, we have corrected our statement of the 0.05Sv, 0.1Sv and 0.2 Sv-FWP experiments to 0.4Sv, 0.8Sv and 1.6 Sv-FWP experiments, respectively.

In this revision work, we added a new experiment with 0.2Sv FWP for 400 model years. As shown in Supplementary Fig. 9, the AMOC strength reduced from the LGM value of ~28 Sv to a weaker state ~20 Sv rather than an 'off-mode' by the 0.2Sv FWP, while there is insignificantly change in the NPIW strength. This suggested that our simulated LGM AMOC state is relatively insensitive to the North Atlantic FWP, thus a stronger hosing is requested to collapse the AMOC, e.g. the 0.4Sv, 0.8Sv and 1.6Sv FWP in this study.

We additionally attached the reanalysis about our previous LGM hosing experiments using a Earth System Model COSMOS. In the COSMOS experiments[53], the 0.2Sv FWP successfully collapsed LGM AMOC to a 'off-mode' (see Supplementary Fig. 9), thus different from the MPI-ESM simulated LGM climate in this study. On the other hand, once the AMOC is weakened, the NPIW formation enhanced together with a stronger intermediate-to-deep ocean stratification, thus in line with the results in this study. This indicated that our findings in this study, i.e. the response of the North Pacific to the 'off-mode' AMOC during HS-1, are valid for the LGM climates with different sensitivity to the North Atlantic freshwater perturbation. This also suggested that our conclusions in this work remain robust after the correction of error about FWP strengths.

In the following, we've done point-by-point response to the reviewers' comments on earlier version of manuscript

Reviewer #1 (Remarks to the Author):

Gong et al. present an intriguing model study documenting changes in North Pacific Intermediate Water (NPIW) formation across HS1. This study addresses an important aspect in paleoceanography, which has been a matter of intense debate during the last decade or so. The model outputs show enhanced NPIW formation during the HS1 concomitant with a substantial reduction in North Atlantic Deep Water (NADW) formation associated with meltwater discharge, consistent with proxy data. Convection in the Sea of Okhotsk and the Bering Sea is enhanced as a result of increasing SSS associated with increased northward transport of saline subtropical waters. Enhanced NPIW formation leads to a strengthening of the intermediate-to-deep stratification, preventing CO_2-rich abyssal waters to communicate with the atmosphere. These observations contradict previous proxy evidence calling for enhanced deep-water formation during HS1. The authors further convincingly show that these observations are robust and are not dependent on the hosing intensity applied to slow down NADW.

I really enjoyed reading the manuscript, although the argumentation can be quite cumbersome in places. It advances our understanding of North Pacific paleoceanography, in particular related to its role in contributing to the deglacial increase in atmospheric CO2. The study has the sort of attributes to warrant publication in a broad audience journal such at Nature Communications. I have listed a number of comments below, which I would like to see addressed before I can recommend this manuscript to be accepted for publication. I hope the authors will find my comments helpful and constructive as they aimed to be.

We thank reviewer #1 for the assessment and comments, which we hope to address satisfactorily as indicated below.

** Please clearly define/quantify what is meant by "abyssal" (l. 14 and throughout), "deep" (l. 19 and throughout) and "intermediate" depths (l. 20 and throughout) as the loose/incoherent definition of these concepts has spurred unnecessary confusion within the community.*

We agree with the reviewer that the 'abyssal water' are not accurately used in the previous manuscript. In the revised version, we have replaced all the 'abyssal water' by 'deep-ocean

water' and given the 'deep-ocean water' a definition of 'the water mass below ~2500m depth', at l. 39.

l.36. This is certainly correct. Please also note that both model (e.g. Schmittner & Galbraith, 2008) and data compilation (Galbraith and Jaccard, 2015) indicate a general weakening of the global efficiency of the biological pump early in the deglaciation, which would have contributed to partition CO2 out of the ocean interior.

We agree with the reviewer that a lower efficiency of the 'biological carbon pump' may induce the net CO_2 outgassing from the ocean during the deglaciation. In the revised manuscript, we've added this point at l.36-37, and included *Schmittner and Galbraith 2008* [ref. 6], *Galbraith and Jaccard 2015* [ref. 7] into reference.

l.41-43. Unclear - please rephrase

we've reworded this part, see l. 43-45.

l. 63-67. Unclear - please rephrase

We've reworded this part, see l. 65-68.

l. 78. The term "suppressed" is confusing here. I would rather say that NADW was "substantially weaker" (consistent with paleoceanographic proxies (e.g. Bradtmiller et al., 2014 NComms; Lippold et al., 2016, EPSL)).

We've changed the word accordingly, see l.82-83. In addition, we've added '*Bradtmiller et al., 2014 Nature Communications*' [ref. 28] and '*Lippold et al., 2016, EPSL*' [ref. 29] into the reference.

l. 84-89. Great! Strong argument in favor of the robustness of the model, highlighting its suitability to address the hypothesis outlined in the introduction.

We thank the reviewer for this comment.

l. 84-87. What are the implications of deeper mixed layer depths for vertical nutrient supply and export production? Proxy evidence in both the Sea of Okhotsk and the Bering Sea indicate lower export production during HS1 (e.g. Brunelle et al., 2010). Is this further evidence for a stronger decoupling between surface and nutrient-rich deep waters?

We agree with the reviewer #1, and our findings support Brunelle et al., 2010 that the deeper mixed layer depths play a role in lowering the export production in the Okhotsk and the Bering Sea during HS-1. More specifically, according to our modelling results, the deepened mixed layer controlled the production export via two physical processes: (1) the deeper mixed layer enhanced the NPIW formation and stronger intermediate-to-deep ocean stratification. This led to a decoupling of surface and intermediate) from the deep ocean, thus limiting the amount of nutrients mixing from the deep into the surface ocean. Consequently, this reduced primary productivity during HS-1. (2) In the upper ocean, the deepened mixed layer of HS-1 also increased the light limitation, by mixing the surface-layer nutrient out of the photic zone. Overall, our modelling results supports the notion in previous studies [e.g. Brunelle et al., 2010] that the deepened mixed layer is a key to explain the coherent occurrence of low export production and low nutrient utilization in the Okhotsk and Bering Sea during HS-1.

l. 121. This argument is consistent with proxy data from covering the entire subarctic Pacific gyre (Kiefer and Kienast, 2005)

We thank the reviewer and have added *Kiefer and Kienast 2005* [ref. 39] into the reference and cited it at l. 158 and 181.

l. 125 – would a stronger Aleutian Low (possibly related to larger North American ice sheets during HS1) increase Ekman pumping in the subarctic Pacific and its marginal seas? If true, this argument would again argue for a stronger decoupling between surface and nutrient-rich deep waters, as export production was consistently lower before the B/A throughout the subarctic Pacific (e.g. Galbraith et al., 2007; Kohfeld & Chase, 2011).

Indeed, our modeling results revealed a strengthening in Aleutian Low when the AMOC weakens, as shown in Fig. 2d and the new Fig. 3a. Notably, the ice sheets in our HS-1 experiments are prescribed the same as those in the LGM simulation (see Methods). Therefore, our modelled strengthening of the Aleutian Low during HS-1 is not related to a larger North American ice sheet. Instead, the stronger Aleutian Low acts as an atmospheric response to the AMOC slowdown. More specifically, this rapid atmospheric teleconnection from the North Atlantic to subarctic Pacific occurs via topical latitudes with higher SSTs in the Eastern Equatorial Pacific acting as a pivot in the mechanism (Supplementary Fig. 4), as discussed in previous modelling studies [ref. 24, 32]. See l. 103-132 in the revised manuscript.

In addition, we agree with the reviewer that the Ekman pumping in the subarctic Pacific became stronger and contributed to the decoupling between surface and deep waters. As shown in new Fig. 3, the subarctic Pacific gyre intensified along with the strengthening in Aleutian Low when AMOC weakened. Dynamically, this intensified Ekman pumping results in a stronger upwelling of relatively warmer, more saline waters from subsurface depths, by enhancing the Ekman pumping effect. In the surface ocean, the thermal buoyancy of these upwelled subsurface waters is counteracted by a heat loss to the cold surface atmosphere; however, these waters remain saline and thus further weaken the surface stratification. This process, together with the stronger advection subtropical-sourced more saline water and reduced net precipitation, acts to intensify the NPIW formation and intermediate-to-deep ocean stratification. We've added the role of 'Ekman pumping' in the revised version, see l. 121-127 in the revised manuscript.

l. 130-131. Does this argument also take into account the upwelling of relatively warmer subsurface waters as a consequence of increased convection during HS1?

We agree with the reviewer that the HS-1 stronger convection of the relatively warmer subsurface waters into the surface ocean may also cause higher SSTs in the Okhotsk and Bering Sea. Therefore, the SST change in the NPIW formation region is a compensation result between the 'atmospheric cooling impact' due to the stronger Aleutian Low and the 'oceanic warming potential' of the following two processes: (1) a stronger advection of subtropical-sourced, relatively warmer water into the subarctic Pacific during HS-1 (2) a stronger upwelling of the subsurface, relatively warmer water into the surface ocean. We changed this part of discussion in the revised manuscript, see l. 158-167.

l. 132. Please also cite Kiefer and Kienast, 2005.

We have done so, see ref. 39.

l. 136. Is there evidence for reduced river discharge during this time interval that would corroborate this argument?

Following the reviewer's suggestion, we searched the literature regarding the terrestrial records surrounding the NW Pacific and its marginal seas, especially from the drainage area of the Amur River that has the largest river discharge into the Okhotsk Sea nowadays. Although there is no direct evidence for winter river discharge around Okhotsk and Bering Seas during HS-1, previous studies have shown qualitative evidence for driver terrestrial conditions around in the

Far East, covering the attachment region of the Amur River, during millennial-scale cold periods of the deglaciation, e.g. Bazarova et al. 2008 [ref. 33] and Mokhova et al. 2009 [ref. 34].

l. 147-163. This argument is confusing and doesn't make sense to me. You mention earlier that NPIW formation increases during HS1 as a result of increasing SSS (while maintaining approximately constant SSTs). As such, I don't understand why NPIW densities decrease compared to the LGM, which is characterized by lower SSS once convection is initiated. I understand that the model requires higher SSS in the Sea of Okhotsk and the Bering Sea to kick-start convection. Does this imply that saline water transported from the subtropical Pacific accumulates in both regions, before initiating convection?
Why then SSS decrease upon initiation of enhanced NPIW formation? Is the northward transport of subtropical waters not efficient enough to replace the volume of water, which has been subducted? In any case this paragraph needs to be clarified.
We agree that this part of statement in the previous manuscript was not clear and thus led to some confusions. Here, we provided detailed explanations in the below and also reworded this paragraph in the revised version accordingly, see l. 192-206.

Our results attribute a stronger NPIW formation to a weakening of the surface halocline on the basis of cold glacial surface ocean and also in favor of a positive 'salinity feedback'. Notably, our simulated stronger NPIW formation in HS-1 occurs on the basis of the continued existence of a halocline between the surface and intermediate ocean since the LGM, although such halocline becomes relatively weaker in HS-1 (Supplementary Fig. 2d-f). At the onset of the stronger NPIW formation during HS-1, the stronger convection transports more surface water with relatively lower salinities down to the intermediate-depth ocean, while it in turn ventilates the relatively more saline water from the intermediate to surface ocean. This process thus works to further weaken the halocline, acting as a positive feedback ('salinity feedback') in the NPIW intensification during HS-1. Moreover, once the NPIW-to-NPDW stratification develops due to the lower salinities of the NPIW during HS-1, it also constrains the impact of relatively colder deep ocean water on the intermediate depths by limiting the vertical water exchange, thus retaining the warmer characteristic within NPIW during HS-1 (Fig. 4 and Supplementary Fig. 2). In turn, this process enhances the deep ocean stratification by enhancing the thermocline between the intermediate and deep ocean, thus creating a positive feedback ('temperature

feedback’, thereafter) in developing an intensified intermediate-to-deep ocean stratification during HS-1.

l. 168 and throughout. Use “Gulf of Alaska” instead of “Alaska Bay”.

We have done so, see l. 216.

Reviewer #2 (Remarks to the Author):

The authors present a modeling study of the early parts of the last deglaciation with a focus on North Pacific Ocean circulation and ventilation changes. The new idea is that enhanced intermediate water formation during HS-1 would advect relatively low salinity waters downward and thus decrease densities of intermediate waters, which would increase the density difference between intermediate and deep waters. I think the authors make a good point that this is a plausible scenario, but I also think that their conclusions and inferences are overconfident and therefore the paper needs major revisions.

We thank reviewer #2 for the detailed comments regarding the fact that our modelling results. In the following, we've made response to each comment.

I appreciate that the authors present sensitivity tests with respect to the amount of freshwater forcing applied to the North Atlantic and atmospheric CO2 concentrations. However, I think the results may also be critically dependent on uncertain initial conditions, which were not considered. E.g. Schmittner et al. (2007, VOL. 22, PA3207, doi:10.1029/2006PA001384) have shown that the response in the North Pacific to North Atlantic hosing depends critically on the background stratification. If the stratification is strong the response is much reduced compared to a weakly stratified initial stratification.

We agree with the reviewer, and indeed our conclusions support the findings by *Schmittner et al. 2007*. Specifically, our modelling results suggested that the surface-ocean halocline in the Okhotsk and Bering Seas is the critical factor that limits the NPIW formation from a higher production rate during the LGM. This thus determines that a weakened halocline is sufficient to initialize a stronger convection and thus a NPIW intensification during HS-1, on the basis of the almost consistently cold (always below 0°C since the LGM) winter surface ocean throughout the LGM and HS-1 climates. At this point, our modelling results are in line with the suggestion of the ocean stratification in controlling the NPIW formation [Schmittner et al. 2007; Menviel et al. 2012]. Here, Both Schmittner et al. 2007 and our modelling results indicate that a more stable LGM ocean with stronger stratification will inevitably requests a higher winter SSS anomalies to collapse the surface halocline to trigger a NPIW intensification. In the revised manuscript, we've included *Schmittner et al. 2007* into our discussion, see l. 139-142 and ref. 37.

Another sensitivity not considered is the initial (LGM) AMOC strength. Currently the LGM AMOC is unknown. Whereas some papers suggest a strong AMOC (Kurahashi-Nakamura et al., 2017,

doi:10.1002/ 2016PA003001) others suggest a weak AMOC (e.g. Schmittner and Somes, 2016, doi:10.1002/2015PA002905). Thus, I think it is fair to conclude that there is no consensus and the LGM AMOC strength remains uncertain. In this context it would be appropriate to discuss the sensitivity of the results to the unknown LGM AMOC strength. E.g. if the LGM AMOC was weak, in contrast to the author's model, the North Pacific response may also be different. I think a discussion of these uncertainties would be appropriate.

We agree with the reviewer that paleoclimate modelling studies showed diverse results about the AMOC strength during the LGM compared to Pre-industrial conditions. Neither marine proxies are able to give an answer. Here, we also agree with the reviewer that the potential impact of the LGM-background AMOC to NPIW response during HS-1 should be addressed in this study. In the revision work, we added a new experiment with a 0.2Sv-FWP by 400yrs firstly and then a stronger hosing of 0.8 Sv by another 120yrs. As shown in the new Supplementary Fig. 9, the 0.2Sv FWP by 400yrs weakens the AMOC from the LGM values of ~28 Sv to a quasi-stable state of ~20 Sv, and then the following 120yr 0.8 Sv hosing successfully collapsed the AMOC to less than 5 Sv, i.e. an 'off mode' state. In parallel, the NPIW formation showed insignificant change due to the 0.2Sv FWP, but it increased significantly from the LGM state to ~2.8 Sv at the 'off-mode' AMOC. Here, we use the LGM experiment and the LGM experiment with 0.2 Sv FWP as represents for the LGM climate with stronger- and weaker-than-PI AMOC, respectively, with the respect to the ~24.5 Sv AMOC in the PI control state (see Supplementary Fig. 1a). Therefore, our experiments showed that the response of the NPIW intensification to the 'off-mode' AMOC is independent from the LGM background with either stronger or weaker AMOC. In the revised version, we added a new discussion paragraph 'Independence of the stronger NPIW formation of HS-1 from the LGM background climate with a stronger or weaker AMOC than the Present Industrial values.' in the Supplementary and added *Schmittner and Somes, 2016* [ref. 63] *Kurahashi-Nakamura et al., 2017* [ref. 64] in the reference.

Previous model results also did not agree on the temperature response in the North Pacific. E.g. Mikolajewicz (1997) and Zhang and Delworth (2005) suggest cooling, whereas others (e.g. Schmittner et al. 2007) suggest warming. I think this also warrants some discussion. If I remember correctly I think Mikolajewicz (1997) suggest an atmospheric mechanism for the cooling, whereas Schmittner et al. (2007) suggest an ocean mechanism (the Atlantic-Pacific seesaw of Saenko et al.). I think the author's experiments could add something to this issue.

We answered this comment together with a following one by the same reviewer that '*125-127 In Mikolajewicz (1997) and Zhang and Delworth (2005) atmospheric …*'.

Here, we agree with the reviewer that previous paleoclimate modelling studies have addressed different response of the North Pacific SSTs to the collapsed AMOC on the basis of the LGM. According to our modelling results, the SST change in the NW Pacific due to the 'off-mode' AMOC results from the competition between the 'atmospheric cooling impact' and 'ocean warming potential'. As discussed in the manuscript: 'atmospheric cooling impact' referred to the stronger Aleutian Low. At the AMOC 'off-mode', the anomalous cyclonic atmospheric circulation of the stronger Aleutian Low transports a larger amount of cold air masses from the East Siberian continent to the Okhotsk and western Bering Seas. This process cools down the regional surface ocean, acting as the 'atmospheric cooling impact'. In parallel, the 'oceanic warming potential' correlates to the stronger northward advection of subtropical warmer water into the high latitudes North Pacific and the upwelling of relatively warmer water from the subsurface layer (due to the stronger ventilation and strengthening of the subarctic Pacific gyre). In the NW Pacific and its marginal seas, the 'atmospheric cooling impact' and 'ocean warming potential' work to change the SSTs in opposite ways, with their competition results determining the ultimate change in the SSTs from the LGM to HS-1. Here, these two competitive processes have important implication in understanding the distinct behaviours of the 'Atlantic-Pacific SST seesaw' in previous modelling studies: when the 'atmospheric cooling impact' acts as the dominant control, the North Pacific shows lower SSTs in HS-1[31, 40, 43]; while when the 'ocean warming potential' becomes in charge, the subarctic Pacific turns to warmer conditions against the cooling in the North Atlantic at the AMOC 'off-mode', thus constituting an 'Atlantic-Pacific thermal seesaw'[37, 41]. In the revised manuscript, we involve added this part of discussion into the main text, see l. 155-181, as well as the following four studies into the reference: *Mikolajewicz 1997* [ref. 42], *Zhang & Delworth 2005* [ref. 40], *Saenko et al. 2004* [ref. 41], *Schmittner et al. 2007* [ref. 37].

98-99 why do NH westerlies intensify? Fig. 3a seem to also show a southward shift. Please comment in text. A map of surface wind or windstress vector anomalies may be useful to see where this intensification happens. I assume much will be over the North Atlantic. Results from Schmittner et al. (2007) indicate that the response in the North Pacific is highly model dependent.

We followed the reviewer's this comment and checked further details in our modelling results. We found that the stronger Westerlies over the North Pacific Ocean is attributed to stronger Aleutian Low system once the AMOC weakened (as shown in the new Fig. 3). This led to anomalous cyclonic circulation over the subarctic Pacific, acting to strengthen the Westerlies at mid latitudes over the North Pacific Ocean. Here, our modelled stronger Aleutian Low system due to the North Atlantic hosing have been commonly revealed in previous modelling studies using different ESMs of with different hosing strengths, and explained by *Wu et al. [2008]* and Okumura et al. [2009]. Moreover explicitly, this rapid atmospheric teleconnection from the North Atlantic to subarctic Pacific occurs via topical latitudes with higher SSTs in the Eastern Equatorial Pacific acting as a pivot in the mechanism (Supplementary Fig. 4), as discussed in previous modelling studies[24, 32]. In the revised manuscript, we have changed this part of discussion, see l. 103-111, and added a new Fig. 3. In addition, we added Supplementary Fig. 4 to show that this mechanism functions in all our experiments with different hosing.

100-101: "As a consequence a larger amount of tropically-sourced saline water is transported northward" This statement is not supported with evidence. How do you know that wind changes are the cause? In some of Schmittner et al.'s (2007) experiments and in Saenko et al. (2004) the North Pacific salinifies as a response to ocean buoyancy forcing only without any wind changes. So, in order to attribute northward salt transport changes to wind a decomposition into overturning and wind-driven gyre transport is needed. Or the statement needs to be changed. Also, how do you know it is "tropically-sourced". I don't see evidence provided for this either. Could it be subtropically-sourced? I'd think that would be more likely.
Because so quick!? Or net Precipitation?
We agree with the reviewer that 'the tropically-sourced saline water' should be 'subtropical-sourced', and have changed this part accordingly in the revised manuscript, see l.61, 112, 155 and 165. We thank the reviewer for this correction.

We also noticed that 'a larger transport of subtropical-sourced saline water into the subarctic Pacific Ocean' via stronger Westerlies is a consequence of the strengthening of Aleutian Low once AMOC weakens. In parallel, the intensified Aleutian Low increases the SSS in the Okhotsk and western Bering Seas via various processes in the atmosphere-ocean coupled system, additionally by (1) reducing the net precipitation over the Okhotsk and western Bering Seas during HS-1; (2) increasing the Ekman pump in the subarctic Pacific gyre region and thus

bringing more subsurface relatively more saline water into the surface ocean. Furthermore, as shown in the new Fig. 3, the strengthening of Aleutian Low, Westerlies over the mid-latitude North Pacific and the subarctic Pacific gyre occurs simultaneously in response to the weakening of AMOC. Thus, as pointed by the reviewer, our previous statement about the wind change as the initial cause of the higher SSS in the NW Pacific is not completely true. Accordingly, we have changed this part in the revised manuscript, see l. 103-132.

109: ")" corresponding opening parenthesis is missing

We've reworded this sentence, see l. 136.

113-114 "wintertime" show seasonal SSS evolution somewhere to back up this statement with evidence

We highlighted the 'wintertime' in this part, because our simulated glacial NPIW formation is a winter process (Supplementary Fig. 3). Here, we also provided the summer SSS anomalies, please see the Fig at the end of this letter.

"extremely cold" SSTs are mentioned e.g. in lines 105, 108 and 205. What does that mean? SSTs can only be at most -2degC cold.

We intended to use the 'extreme cold SSTs' for a cold surface ocean lower than 0°C in the previous manuscript. We've noticed that "extremely cold" SSTs is an improper statement. Therefore, we've reworded this part throughout the revised manuscript, see l. 136-137,153 and 257.

109-110 "that a presence of relatively stronger surface halocline in LGM (Supplementary Fig. 2)" stronger compared to what? The pre-industrial control? But this is not shown in Fig. S2. Again, evidence seems to be missing to support this statement. Also, an explanation as to why the halocline was stronger would be useful. Is it because of the closure of Bering Strait?

We apologized for the unclearness in the previous manuscript. In this part, we actually intend to argue our modelling feature that the surface halocline remained in the LGM, as that in the PI simulation. On the other hand, our LGM experiment revealed a stronger NPIW formation than that in the PI simulation (Supplementary Fig. 1), in line with marine proxy evidences [e.g. Keigwin 1998]. In the revised manuscript, we've changed this part, see l.139-140.

125-127 In Mikolajewicz (1997) and Zhang and Delworth (2005) atmospheric circulation changes lead to cooling. See also discussion in paragraph 18 of Schmittner et al. (2007). So, models don't seem to show consistent responses in the North Pacific due to an AMOC shutdown.

We answered this comment together with the previous one from the same reviewer that '*Previous model results also did not agree on the temperature response in the North Pacific …*'.

129: "tropical" see above

We changed 'tropical' to 'subtropical' in the revised version, see l. 165. Thank you for the correction.

144: "act" syntax; I suggest "act as"

We've reformulated the entire sentence, see l. 184-187.

169: I'm not sure I see a "stronger southward flux of lateral salt transport" in the figure. The figure shows positive and negative patterns. Please refer to specific features in the figure. Also, why only show the eastern portion of the basin? Why not show the whole zonal section? Surely the western part also transports salt.

Following the reviewer's comment, we've calculated the T, S, V, TxV and SxV transect at 53°N across the entire North Pacific Ocean, as shown in the new Fig. 4a. By the meantime, we also provided the detailed change of T, S, V, TxV and SxV in Supplementary Fig. 6. As shown in Fig. 4a, there is stronger northward flux of lateral salt transport in the upper 500m, while a larger southward flux of lateral salt transport in the intermediate ocean.

171: "stronger southward ocean flow" do you mean the in Fig. 5b between 130W and 140W? It would be helpful for the reader to point out specific features as precisely as possible, otherwise the reader is forced to guess what is meant.

According the reviewer's previous comment, we've calculated the salt flux at 53°N across the entire North Pacific Ocean, please see the new Fig. 4a.

172: "thus a depict" syntax

We've changed the wording, see l. 213-215.

191 "HS-1, higher CO2" syntax; I suggest "higher CO2 during HS-1"

We've changed accordingly, see l. 240.

206 "and are" syntax

We've changed the wording, see l. 254-258.

220 Since ventilation ages were not simulated attribution of their changes is not possible. This is speculation.

We agree with reviewer and changed this argument to a suggestion on the basis of our modelling results, see l. 273-275.

225-226 "rule out" I don't think this can be ruled out. I think the authors present a physically consistent viable mechanism but because no real apples-to-apples data model comparison has been performed ruling out other scenarios is not possible.

We agree with the reviewer that this part of our argument is too strong by excluding other physical controls. For instance, a recent study of Du et al. NatureGeo 2018 [ref. 13] has presented an opposite view. Therefore, more palaeoceanographic evidences in future study are required for better understanding about the North Pacific circulation during the last deglaciation. In the revised manuscript, we reworded this argument, see l. 277-279.

Reviewer #3 (Remarks to the Author):

This manuscript presents interesting model results about how North Pacific stratification responded to circulation changes during Heinrich 1 with implications for better understanding the source and timing of carbon release from the deep ocean to the atmosphere during the deglaciation. This topic is actively debated among researchers and is likely to be of interest to a relatively broad audience. Hence, it may be suitable for publication in Nature Communications after revision. I have several concerns about the manuscript's current form that could be addressed with major revisions. Generally, the manuscript needs to provide more background about previous studies and more detail about the model results and uncertainties. Also, the manuscript needs revision to improve the writing and figure presentation.

We thank reviewer #3 for the assessment and constructive comments. In our revision work, we have addressed our reply to all the comments and changed the manuscript accordingly.

Placing the new results in the context of previous research:

1. This manuscript should compare the new results with previous modeling efforts about this question (e.g., Chikamoto et al., 2012). How do the current modeling results compare with previous ones? How do differences between models potentially contribute to differences in the results?

According to the reviewer's comment, we have made further comparison of our results to previous paleoclimate modelling work (see l. 106-109, 170-181), and totally 15 additional references have been included in the discussion of the revised manuscript.

Here, we agree with the reviewer that *Chikamoto et al., 2012* is a milestone work that explored the understanding of the glacial NPIW formation. Both their results and this study commonly presented the coherence between the strengthening of NPIW formation and the intensification of the subarctic Pacific overturning circulation. Moreover, compared to *Chikamoto et al., 2012*, our modelling work revealed that the glacial NPIW formed in both the Sea of Okhotsk and Bering Sea thus in line with marine proxies. This was not solved in *Chikamoto et al., 2012*, likely due to the lower resolution in their ocean model. On the other hand, although distinct from the Chikamoto et al., 2012 that colder surface ocean in the NW Pacific is key to enhance the NPIW formation during HS-1, our results suggested a weakening rather than completely removal *[Menviel et al. 2012]* of the surface halocline plays the dominant role in determining the stronger NPIW formation of HS-1, see l. 150-154.

In particular, our modelling results provides a unifying explanation for the distinct behaviours of the 'Atlantic-Pacific SST seesaw' in previous modelling studies: when the 'atmospheric cooling impact' acts as the dominant control, the North Pacific shows lower SSTs in HS-1[31, 40, 43]; while when the 'ocean warming potential' becomes in charge, the subarctic Pacific turns to warmer conditions against the cooling in the North Atlantic at the AMOC 'off-mode', thus constituting an 'Atlantic-Pacific thermal seesaw'[37, 41]. In this study, our results provide a case of close contest between the 'atmospheric cooling impact' and 'oceanic warming potential'. This determines the SSTs of HS-1 to be overall comparable with the LGM values in the Okhotsk Sea (Fig. 2a), explaining the same climatic signals in the paleo temperature reconstructions from this region[38, 39].

2. Lines 132 and 145 claims that the new model results agree well with paleo-temperature reconstructions from proxies. The manuscript needs a figure (or supplemental figure) that presents a comparison of the proxy and model results.

Riethdorf et al. 2013 and *Kiefer and Kienast 2005* characterized comparable SSTs in the NW Pacific throughout the LGM to HS-1 climates on the basis of a series of paleo-temperature constructions, in line with our modelling results as shown in Fig. 2a. Notably, these paleo-temperature constructions are on the basis of multiple marine proxies. The conclusion about the comparable-with-LGM SSTs during HS-1 are drawn on the basis of a well consideration of the backgrounds (e.g. seasonal and living habitat depths) of these marine proxies and their relationship with SST signals. In the revised manuscript, we've included both *Riethdorf et al. [2013]* and *Kiefer and Kienast [2005]* into the reference see ref. 38 and 39, and cited in the l. 155-158, 178-181. We believe that their discussion about the marine proxies and their climatic indications are necessary to show the in-line between our modelling results and data.

3. Line 54 lists sea ice expansion as a potentially important mechanism for affecting NPIW formation during HS-1. However, this appears to be the only mention of sea ice in the entire manuscript. The authors need to provide information about any sea ice changes that occurred in their model and how these did or did not contribute to NPIW formation change.

We agree with the reviewer that the sea ice response along the stronger NPIW formation to the AMOC slow-down during HS-1 should be discussed. Therefore, we've added one paragraph and one figure in the Supplementary of the revised manuscript. As shown in Fig. 2a and

Supplementary Fig. 10, the winter sea ice concentration became relatively less compared to the LGM conditions in the Sea of Okhotsk and Bering Sea, in response to the regional relatively higher but still lower-than-0°C SSTs due to the stronger advection of the subtropical relatively warmer water when AMOC weakened (Fig. 2a). On the other hand, in spite of the sea ice melting, the SSS in the Okhotsk and Bering Seas became higher due to the stronger advected subtropical water with relatively higher salinities. This suggested that the winter sea ice no longer played a dominant role in changing winter SSS and thus surface halocline on the basis the maximal glacial conditions, thus not the key factor in determining the stronger NPIW formation of HS-1. Here, the modelling simulation of HS-1 less sea ice and relatively higher SSTs compared to the LGM conditions were also indicated in previous studies [e.g. Saenko et al., 2004; Schmittner et al., 2007; Okazaki et al. 2010; Menviel et al. 2012]. Moreover, although sea ice reduced in our HS-1 experiment, our modelled HS-1 sea ice edge remained in line with the sea ice coverage region on the basis of PIP25 evidences [Max et al. 2012; Méheust 2018] (Supplementary Fig. 10e, f). In the revised manuscript, we've added the supplementary paragraph 'Response of the NW Pacific sea ice to 'off-mode' AMOC' and Supplementary Fig. 10.

4. Additionally, the authors should discuss how their model compares with other Earth System model results for the pre-industrial and LGM (particularly with respect to overturning strength/depth and sea ice extent). Could model differences contribute to any bias in the results?

We agree with reviewer that the simulations of LGM-to-PI climate anomalies are different among various ESMs, especially in sea ice and AMOC change. In the following, we provided our reply to this comment from two aspects:

(1) Independence of our modelling results from the LGM background with either stronger or weaker AMOC compared to PI conditions. Previous modelling studies showed diverse results about the AMOC strength of the LGM either stronger or weaker than the pre-industrial conditions, e.g. Kurahashi-Nakamura et al. 2017 and therein. As shown in Supplementary Fig. 1a, c, our LGM experiment presented relatively stronger AMOC compared to the pre-industrial state with comparable depths in the AMOC cell. In the revision work, we made a new experiment with a 0.2Sv-FWP by 400yrs firstly and then a stronger hosing of 0.8 Sv by another 120yrs, As shown in Supplementary Fig. 9. In this new experiment, the 0.2Sv FWP by 400yrs

weakens the AMOC from the LGM values of ~28 Sv to a state of ~20 Sv, and then the following 120yr 0.8 Sv hosing successfully collapsed the AMOC to less than 5 Sv, i.e. an 'off mode' state. In parallel, the NPIW formation showed insignificant change due to the 0.2Sv FWP, but it increased significantly from the LGM state to ~2.8 Sv at the 'off-mode' AMOC. Here, we use the LGM experiment and the LGM experiment with 0.2 Sv FWP as represents for the LGM climate with stronger- and weaker-than-PI AMOC, respectively, with the respect to the ~24.5 Sv AMOC in the PI control state (see Supplementary Fig. 1a). Therefore, our experiments showed that the response of the NPIW intensification to the 'off-mode' AMOC is independent from the LGM background with either stronger or weaker AMOC. In the revised version, we added a new discussion paragraph in the Supplementary together with Supplementary Fig. 9.

(2) Advanced sea ice in our LGM simulation compared to the Pre-industrial climate. Previous modelling studies have commonly suggested sea ice growth from the pre-industrial to LGM state by distinct amount and spatial characteristics, although there were differences in AMOC strengths. Similarly, our LGM also revealed more sea ice from the PI to LGM conditions (Supplementary Fig. 11). Moreover, the PI-to-LGM growth of sea ice in our modelling results are neither extreme high nor low, but a moderate growth conditions compared to other ESM simulations. Therefore, our findings about the NPIW change from the LGM to HS-1 climate are independent from the background AMOC and not correlated to extreme conditions of sea ice coverage in the LGM state.

5. How long was the spin-up for the LGM run? What condition was used to determine when the LGM had reached a "quasi-equilibrium state"?
We've added this information in the Methods, see l. 20-22. Here, our LGM experiment simulates for 4000 model years until a quasi-equilibrium state with globally averaged SST change less than 0.05°C/100yr and no trend in the variability of the AMOC strength.

Writing and figure presentation:
6. There are quite a few sentences in this manuscript that are awkwardly or incorrectly phrased. The manuscript needs extensive revision with this in mind.
For example, lines 41-45.
We've changed the wording, see l. 43-45.

A few other examples of word choice problems:

- Line 46-48: I wouldn't describe a change in ventilation as a "fundamental physical characteristic."

We've changed the wording, see l. 48-51.

- Lines 64-66: "a hypothesized overturning ... CONTRASTS (instead of contrasted) with a decoupling …and CONTINUED (instead of remained) isolation …"

We've changed this sentence, see l. 65-68. Thanks!

- Line 172: "thus a depict of stronger influence" ???

We've changed the rewording, see l. 213-215.

- Line 378: "radish colours present a stronger northward transport" should be "red colours represent …"

We've changed the original Fig. 3a to the Supplementary. In the revised version, we've reworded this part, see the caption of Supplementary Fig. 6.

7. Because the abstract begins with a question about CO2 release from the North Pacific, it would be nice if it ended with a more definitive statement about when/how this CO2 release occurred (instead of only a statement about prolonged isolation of the deep Pacific). The manuscript addresses this in more detail but the abstract feels incomplete.

We agree with the reviewer that the exact time for the release of the North Pacific benthic carbon during the last deglaciation is an important point. For instance, *Gray et al. 2018 Nature Geoscience* [ref. 10] recently suggested a mechanism that a stronger upwelling in the subpolar North Pacific worked together with collapsed NPIW formation at the onset of the Bølling–Allerød, leading to CO_2 outgassing from the North Pacific Ocean. On the other hand, our work in this study aims to explore the understand why the North Pacific deep-ocean carbon has a constrained contribution to the rapid increase of the atmospheric CO2 during the HS-1. In the revised manuscript, we modified the abstract on the basis of our modelling results that the CO_2 would be released after the stronger NPIW formation of HS-1 (see l. 27-29), and included *Gray et al. 2018* into the main text (see l. 40-43) as ref. 10.

8. Figure 1f: I recommend flipping the y-axis to match the sense of depth increasing downward.

We thank the reviewer for this comment to help with the figure. By comparing the figure before and after flipping the y-axis, we think that the current figure has its own advantage in helping the readers to understand our modelling signals, for the following reason: in the current figure, the curves 'e' and 'f' go up together, clearly indicating that the stronger NPIW formation changed along with the deeper mixed layer in the Sea of Okhotsk and Bering Sea.

9. Figure 3: These are three unrelated subplots that shouldn't be grouped together. I recommend keeping only one of these subplots in the manuscript and changing the rest to supplemental figures.

We agree with the reviewer about the complexity of the previous Fig. 3. In the manuscript, we have reformed the previous Fig. 3 to the new Fig. 4. Now, the Fig. 4 focus on explaining the vertical profile change in the North Pacific Ocean and in a neat way.

10. Figure 4: I think the colors here illustrate an absolute scale for salinity/density, but the caption makes it sound like the colors indicate change relative to some reference: "… colors indicate an increase in salinities …"

We've changed accordingly in the revised manuscript, see the caption of the new Fig. 5. Thanks for correcting us!

11. It's confusing that Figures S1 (b, d and f) have a different y-axis range than S1 panels a, c, and e. It would also be nice if S1 (b, d and f) matched the same x and y axis ranges as Figure S2. Figures 3b and S5 could also match the vertical scale of these other plots.

We apologized for the inconsistent depth range in previous figures. In the revised manuscript, we've commonly used 0-4000m depth range for Fig. 4, Supplementary Fig. 2 and Supplementary Fig. 6, while we still kept Supplementary Fig. 1 in a zoom-in view of the upper 3000m depths to highlight the NPIW features. Thank you!

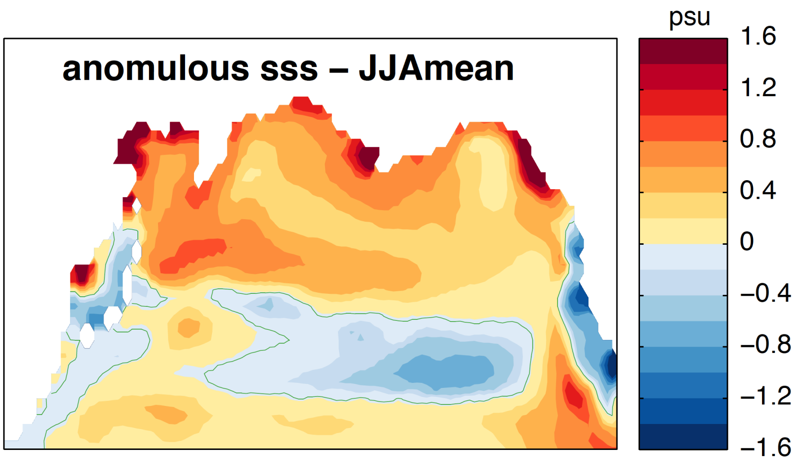

Fig. Modelled North Pacific summer SSS change due to the AMOC slow-down.

Reviewer #2 (Remarks to the Author):

In response to my previous point about the initial LGM AMOC state the authors included an additional experiment and discus it in the Supplementary Material section "Independence of the NPDW intensification of HS-1 from the LGM background AMOC and the dominant control of the 'off-mode' AMOC". In this experiment the LGM AMOC is reduced from 28 Sv to 20 Sv. However, I think that this still is an unrealistic initial state. Muglia et al. (2018) show that the LGM AMOC was most likely much weaker (~8 Sv). So, my question is: how would this bias in the initial state affect the authors' results. I think this is a major caveat and has to be discussed in the main text.

Line 110: remove "a"

Muglia, J., L. C. Skinner, and A. Schmittner (2018), Weak overturning circulation and high Southern Ocean nutrient utilization maximized glacial ocean carbon, Earth Planet Sc Lett, 496, 47-56, doi: 10.1016/j.epsl.2018.05.038.

Reviewer #3 (Remarks to the Author):

This revised manuscript is much improved. My remaining concerns are primarily about clarifying some of the explanations and improving the writing in a few places.

1. The most important areas for clarification are the discussions of various Atlantic-Pacific seesaws. First, the terminology needs to be made consistent. I am assuming that the terms "SST seesaw" and "thermal seesaw" are supposed to be the same. Similarly, "MOC seesaw" and "overturning seesaw" seem to be used interchangeably. I find the discussion of the MOC/overturning seesaw particularly problematic. I think the term should be defined more precisely with regards to whether the authors are using it to refer to intermediate-depth overturning or deep overturning. The citations they provide seem to be mostly focused on deep convection. In contrast, this manuscript says there isn't a deep Atlantic-Pacific seesaw, but that only NPIW overturning increases when AMOC decreases. This would be the opposite of lines 248-249 which seems to support the original notion of a deep overturning seesaw.

2. Line 177: If the balance between atmospheric cooling and ocean warming is a "close contest," how can you be sure that your model result is robust?

3. Lines 210 and 212: I don't understand how NPIW change provides "lower salinities" to lower latitudes (I assume this refers to intermediate depths?) if there is "stronger-than-LGM southward lateral salt flux at intermediate depths." These two things seem to be contradictory.

4. Line 236: I don't understand the statement that "NPIW formation is quantitatively independent from the AMOC slow-down process." In both models the "slow-down process" is fresh water flux (just a different amount). Also, what is meant by "quantitatively independent"?

5. The phrase "on the basis of" is used frequently throughout this manuscript and replies to reviewers, but I'm not sure what it is intended to mean. Sometimes it seems to me "in comparison to" and sometimes it seems to mean "in conjunction with." I recommend replacing all instances of "on the basis of" because it is ambiguous and awkward.

6. Lines 66-68: awkward phrasing: " … there is hypothesized overturning down to the deep ocean of ~3000 m, in contrast with a decoupling of the deep from mid-depth (~ 2000m depth) water and a continued isolation of the NPDW during HS-1"

7. Line 45: "Once down to the deep ocean is established" missing the word "convection"

8. Line 107: "topical" should be "tropical"

9. Line 119: "driver" should be "drier"?

10. Line 135: initialized should be initiated?

11. Why not say "similar to" instead of "not significantly colder than"?

12. Line 246: Is this the correct figure? The caption for Supplemental Figure 6 doesn't mention anything about a different CO2 concentration. (Also, the word "Figure" is missing.)

13. Line 273: It is confusing that you say deep Pacific ventilation age increases when there is basically no change in NPDW age shown in Figure 1. What accounts for this difference? For example, I noticed that citation 25 uses a core from the NE Pacific whereas Fig 1 is data from the NW Pacific. Does the model predict a difference between the NE and NW? In any event, this discrepancy or difference should explained.

14. Line 286: awkward phrase: “to increase the understanding of the physical determinants of …”

15. Caption for Suppl. Fig. 1 doesn’t list all the subpanels.

In the following, we've done point-by-point response to the reviewers' comments on earlier version of manuscript

Reviewer #1 (Remarks to the Author):

The authors have done a very thorough job addressing the reviewers' comments. The manuscript has improved quite substantially and I feel is now acceptable for publication in Nature Communications. Congratulations!

We thank reviewer #1 for the assessment and all the comments.

Reviewer #2 (Remarks to the Author):

We thank reviewer #2 for the comments to our manuscript.

In response to my previous point about the initial LGM AMOC state the authors included an additional experiment and discus it in the Supplementary Material section "Independence of the NPDW intensification of HS-1 from the LGM background AMOC and the dominant control of the 'off-mode' AMOC". In this experiment the LGM AMOC is reduced from 28 Sv to 20 Sv. However, I think that this still is an unrealistic initial state. Muglia et al. (2018) show that the LGM AMOC was most likely much weaker (~8 Sv). So, my question is: how would this bias in the initial state affect the authors' results. I think this is a major caveat and has to be discussed in the main text. -- Muglia, J., L. C. Skinner, and A. Schmittner (2018), Weak overturning circulation and high Southern Ocean nutrient utilization maximized glacial ocean carbon, Earth Planet Sc Lett, 496, 47-56, doi: 10.1016/j.epsl.2018.05.038. --

Following your suggestion, we have implemented the recent work of Muglia et al. 2018 in EPSL (new ref. 49 in the main manuscript) to represent the spread of potential solutions for the AMOC strength in the LGM state. In Muglia et al. 2018 the weak LGM AMOC state has been simulated by reducing the southern hemisphere's meridional moisture transport almost to the 'low-end' in their UVic model to tolerate an active AMOC under the LGM forcing. Besides the experimental set-up, this study also relies on a different model complexity and model physics. To reflect this in the revised main text we have included the following text (l. 237-243):

Furthermore, we have performed an additional experiment with a stepwise increase in freshwater perturbation (Supplementary Fig. 9). This experiment shows that the North Pacific responses to the AMOC 'off-mode' is robust for basic LGM AMOC background states that can be weaker or stronger than in our simulated Pre-industrial state. These background strengths are comparable to other estimates for LGM AMOC states [Otto-Bliesner et al. 2007 (ref. 47); Muglia & Schmittner 2015 (ref. 48)], although much weaker basic states [Muglia et al. 2018 (ref. 49)] cannot be excluded.

Line 110: remove "a"

We have done so, see l. 111. Thanks!

Reviewer #3 (Remarks to the Author):

This revised manuscript is much improved. My remaining concerns are primarily about clarifying some of the explanations and improving the writing in a few places.

We thank reviewer #3 for the positive appraisal and all the help with our manuscript.

1. The most important areas for clarification are the discussions of various Atlantic-Pacific seesaws. First, the terminology needs to be made consistent. I am assuming that the terms "SST seesaw" and "thermal seesaw" are supposed to be the same. Similarly, "MOC seesaw" and "overturning seesaw" seem to be used interchangeably. I find the discussion of the MOC/overturning seesaw particularly problematic. I think the term should be defined more precisely with regards to whether the authors are using it to refer to intermediate-depth overturning or deep overturning. The citations they provide seem to be mostly focused on deep convection. In contrast, this manuscript says there isn't a deep Atlantic-Pacific seesaw, but that only NPIW overturning increases when AMOC decreases. This would be the opposite of lines 248-249 which seems to support the original notion of a deep overturning seesaw.

We fully agree with the reviewer that we should use the same terminology for one pattern. In the revised manuscript, we've replaced all 'SST seesaw' by uniformly using 'thermal seesaw'. Similarly, we've also replaced all 'overturning seesaw' by 'MOC seesaw'. In addition, following the reviewer's suggestion, we've precisely defined that the 'Atlantic-Pacific MOC seesaw' in our study refers to the response of stronger NPIW formation to AMOC 'off-mode', and also adjusted the citation of references in this part of the manuscript with the focus on 'AMOC *vs.* NPIW'. Please see l. 173 and 253-256 in the revised manuscript.

2. Line 177: If the balance between atmospheric cooling and ocean warming is a "close contest," how can you be sure that your model result is robust?

Here, we use 'close contest' to describe our modelling results that the 'atmospheric cooling' and 'ocean warming' results in overall similar SSTs in the NW Pacific during HS-1 compared to the LGM state. Actually, this part of our results enhances the robustness of our inference about a role of the weakened surface halocline in initiating the NPIW intensification during HS-1, by constraining the thermal potential of a colder surface ocean. Moreover, the 'close contest' of our modelling results is in line with paleoceanographic reconstructions of the comparable surface ocean temperatures between the LGM and HS-1 in the Okhotsk Sea (ref. 38 and 39). In the revised version, we have rephrased this sentence for better illustration, please see l. 177-180.

3. Lines 210 and 212: I don't understand how NPIW change provides "lower salinities" to lower latitudes (I assume this refers to intermediate depths?) if there is "stronger-than-LGM southward lateral salt flux at intermediate depths." These two things seem to be contradictory.

As pointed by the reviewer, this sentence has been misworded in previous version. We've corrected 'stronger-than-LGM southward lateral salt flux' to 'stronger-than-LGM southward transport of low-salinity water at the intermediate depths' in the revised manuscript, please see l. 211-213. Thank you for correcting us.

4. Line 236: I don't understand the statement that "NPIW formation is quantitatively independent from the AMOC slow-down process." In both models the "slow-down process" is fresh water flux (just a different amount). Also, what is meant by "quantitatively independent"?

Here, we intended to use 'quantitatively independent' to describe our modelling results that the NPIW formation became intensified at the AMOC 'off-mode' regardless the speed of AMOC slow-down processes, although a stronger freshwater perturbation collapses AMOC in a faster way and results in a further intensification of NPIW. In the revised version, we have rephrased this sentence for clearer illustration, please see l. 234-237.

5. The phrase "on the basis of" is used frequently throughout this manuscript and replies to reviewers, but I'm not sure what it is intended to mean. Sometimes it seems to me "in comparison to" and sometimes it seems to mean "in conjunction with." I recommend replacing all instances of "on the basis of" because it is ambiguous and awkward.

We fully agree with the reviewer that 'on the basis of' should be replaced by more accurate phases. Indeed, as suggested by the reviewer, 'in conjunction with' is a better choice to illustrate the combined effect of 'cold glacial surface ocean' and 'weakened surface halocline' in initiating the stronger NPIW formation during HS-1. Therefore, we've changed all 'on the basis of' to 'in conjunction with' if necessary throughout the revised manuscript, see l. 152-153 and l. 264.

6. Lines 66-68: awkward phrasing: "… there is hypothesized overturning down to the deep ocean of ~3000 m, in contrast with a decoupling of the deep from mid-depth (~ 2000m depth) water and a continued isolation of the NPDW during HS-1"

We have changed the wording, see l. 65-69.

7. Line 45: “Once down to the deep ocean is established” missing the word “convection”

We have done so, see l. 45. Thank you!

8. Line 107: “topical” should be “tropical”

We have done so, see l. 108.

9. Line 119: “driver” should be “drier”?

We have done so, see l. 120.

10. Line 135: initialized should be initiated?

We have done so, see l. 135.

11. Why not say “similar to” instead of “not significantly colder than”?

We have done so, see l. 136.

12. Line 246: Is this the correct figure? The caption for Supplemental Figure 6 doesn’t mention anything about a different CO2 concentration. (Also, the word “Figure” is missing.)

We have added the missing part in the supplementary figure caption and rechecked all the figure citation numbers throughout the revised manuscript. Thanks!

13. Line 273: It is confusing that you say deep Pacific ventilation age increases when there is basically no change in NPDW age shown in Figure 1. What accounts for this difference? For example, I noticed that citation 25 uses a core from the NE Pacific whereas Fig 1 is data from the NW Pacific. Does the model predict a difference between the NE and NW? In any event, this discrepancy or difference should be explained.

We agree with the reviewer that the paleoceanographic results in Fig. 1c and Lund et al. 2011 (ref. 25) showed discrepancies between each other, although both of these two literatures measured the ventilation ages from the subarctic Pacific. Here, Fig.1c shows stacked records based on a compilation of radio-carbon data collected from the NW Pacific including the Okhotsk and Bering Seas, while Lund et al. 2011 has measured a single core from NE Pacific. In comparison, the stacked curves in Fig. 1c are based on larger dataset and, more importantly, from a region covering the NPIW formation area. Specifically, a few data points belonging to the NPDW stacked curve in Fig. 1c also show older-than-LGM ventilation ages during HS-1 like that

in Lund et al. 2015, while the stacked curve based on the entire dataset shows a universal signal of no significant NPDW rejuvenation in the NW Pacific. Paleoceanographic studies have attributed the measured NW-NE Pacific discrepancies in ventilation ages (e.g. between Fig.1c and Lund et al. 2015) to potentially localized vertical processes and their integration along the way of NPIW transport out of the NW Pacific, as discussed in ref. 10 and 11. Following the reviewer's suggestion, hereby we provided a figure that shows our simulation of the vertical stratification change in the NW, Central and NE Pacific due to the AMOC slow-down (please see the figure at the end of this letter). As shown, our modelling results suggested that the stronger NPIW formation in the NW Pacific enhanced the intermediate-to-deep ocean stratification in both NW and NE Pacific during HS-1. Here, in spite of some discrepancies, both NW (Fig.1c) and NE Pacific (Lund et al. 2015) have commonly demonstrated that there is no significantly rejuvenation in NPDW while the NPIW ventilation age rapidly decreased during HS-1, suggesting a decoupling of NPDW from NPIW during HS-1. Therefore, both these two geological datasets support our findings in this work. In the revised version, we've included both Max et al. 2014 (ref.15 for Fig.1c) and Lund et al. 2011 (ref. 25) to highlight the alignment between our modelling findings and the indications by the two paleoceanographic datasets, see l. 278-280.

14. Line 286: awkward phrase: "to increase the understanding of the physical determinants of …"

We have changed the wording, please see l. 294.

15. Caption for Suppl. Fig. 1 doesn't list all the subpanels.

We have added the missing part of the caption for Supplementary Fig. 1. Thanks!

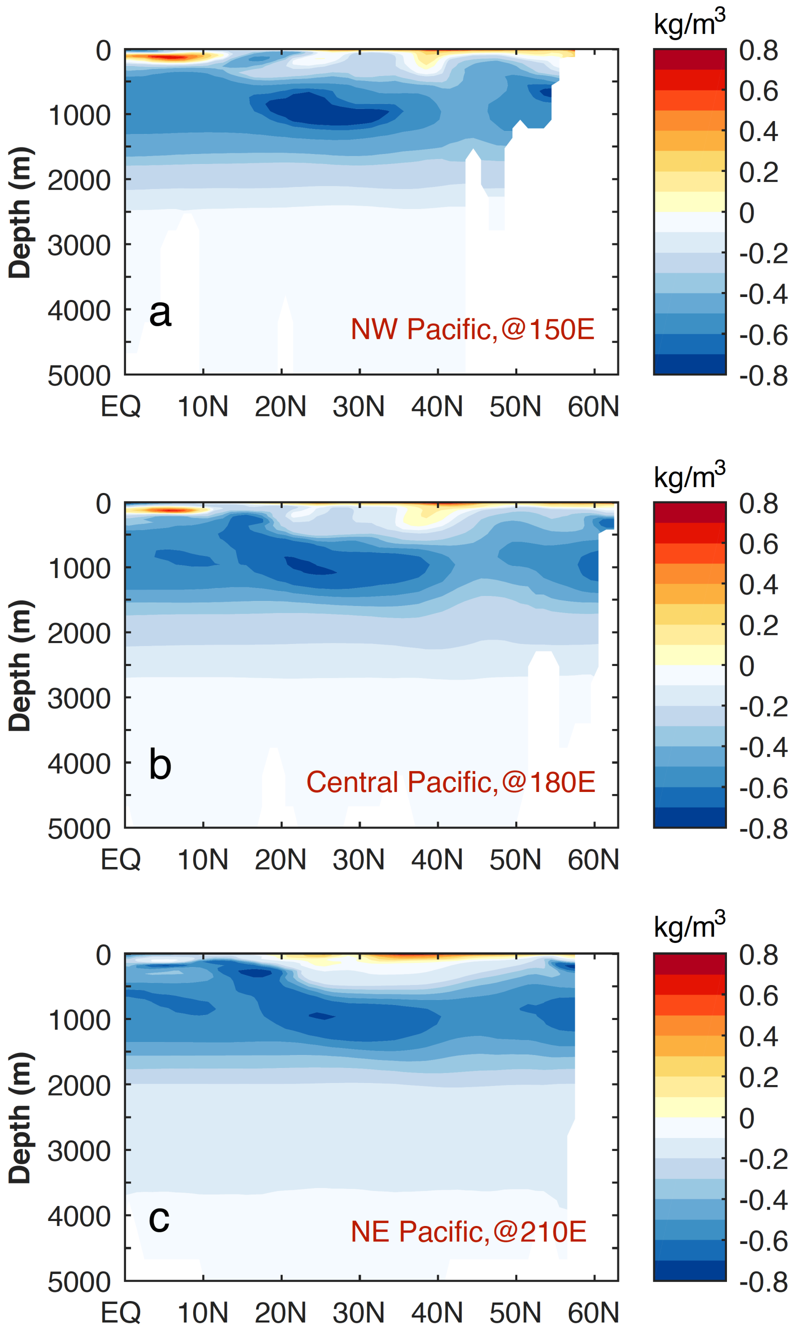

Fig. Modelled density change in the North Pacific due to the AMOC slow-down. a. along 150°E (NW Pacific); b. along 180°E (Central Pacific); c. along 210°E (NE Pacific).
